# X‐ray and γ‐ray Sensing from Aqueous‐Based Lead Sulfide Telluride Nanocomposites

**DOI:** 10.1002/smll.202504684

**Published:** 2025-10-25

**Authors:** Vinh‐Dien Le, Drew A. Vecchio, Ayse D. Uyulur, Ill‐hyuk Han, Andrei M. Ursu, Geehyun Kim, Mark D. Hammig

**Affiliations:** ^1^ Department of Chemical Engineering University of Michigan Ann Arbor MI 48109 USA; ^2^ Department of Nuclear Engineering and Radiological Sciences University of Michigan Ann Arbor MI 48109 USA; ^3^ Amphionic LLC Plymouth MI 48170 USA; ^4^ Nuclear Engineering Seoul National University Seoul 08826 South Korea

**Keywords:** lead sulfide telluride, nanosynthesis, nuclear radiation sensors, radiation shielding, X‐ray and γ‐ray detection

## Abstract

Lead chalcogenides (PbS, PbSe, and PbTe) are exceptional semiconductors for a variety of applications that exploit their unique combination of heavy atomic mass and high charge mobility. For sensors of energetic quanta such as X‐rays and γ‐rays, the de‐coupling of electronic and phononic modes can deliver high intrinsic energy resolution at room temperature. Thus far, the lack of a simple, effective fabrication procedure for making large‐volume percolating networks has prevented the exploitation of these materials. Here, a synthesis strategy is reported that utilizes aqueous colloids of PbS_x_Te_y_ nanoparticles that are grafted upon an aramid nanofiber scaffold, such that excellent charge transport is realized throughout the size‐scalable solids. When sensing *individual* X‐rays and γ‐rays, the resulting nanoparticle‐polymeric composite can exhibit an energy resolution equivalent to that of a commercial single‐crystalline cadmium telluride detector (2.8 keV at 81 keV). Furthermore, the addition of interfaces and randomly distributed lattice planes within the solid results in enhanced stopping of the secondary electrons from the photonic interactions, a scattering effect that can result in either thin‐film X‐ray stopping layers or lightweight, flexible, charged‐particle shielding.

## Graphical abstract

PbSxTey nanoparticles integrate into flexible, size‐scalable aramid polymeric scaffolds, forming percolation paths capable of efficiently transporting the electrons and holes created by penetrating photonic interactions. The resulting nanosemiconductor‐nanofiber composite forms the basis for room‐temperature spectroscopic sensors of X‐rays and γ‐rays that deliver energy resolutions comparable to single‐crystalline CdTe detectors.

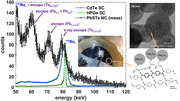

## 1 Introduction

Materials that are developed for sensing optical photons and other quanta interacting in the near‐surface region can tolerate atomic disorder or polycrystallinity because the limited mobility‐lifetime products of the carriers can be compensated with thin active layers and grain boundary passivation.^[^ ^1^, ^2^, ^3^ ^]^ For instance, for the *current‐mode* sensing of X‐ray and γ‐ray fluences, polycrystalline and amorphous materials, such as stabilized amorphous selenium,^[^ ^4^, ^5^, ^6^, ^7^ ^]^ lead‐oxide,^[^ ^4^, ^8^, ^9^, ^10^ ^]^ and amorphous silicon^[^ ^11^, ^12^, ^13^, ^14^, ^15^ ^]^ have been developed and commercialized. For *pulse‐mode* sensing, in which the energy of each quanta is measured, polycrystalline solution‐grown halide perovskites, operating at zero volts, have been shown to exhibit equivalent performance to photomultiplier tubes when sensing scintillation photon pulses following γ‐ray interactions.^[^ ^11^, ^16^, ^17^, ^18^ ^]^ However, semiconducting materials utilized for the direct sensing of highly penetrating quanta, such as γ‐rays, are almost exclusively made from single‐crystalline solids because crystalline order with minimal defects is required to efficiently collect the charge carriers created over the thick (≈1 cm) active regions used to realize acceptable detection efficiency. We show that although interfaces and defects are typically associated with charge trapping, one can purposefully introduce multitudinous boundaries within the nanosolid and achieve spectroscopic performance similar to single‐crystalline materials. Furthermore, although voids are typically viewed as imperfections to be prevented, we show that the addition of nanoscale interfaces results in the more effective capture of the secondary electron information.

Our innovation focuses on the spectroscopic detection of individual high‐energy photons – so‐called *pulse‐mode* radiation detection, rather than the current induced by an integrated X‐ray flux incident upon the readout plane, termed *current‐mode* sensing. For the latter, there have been several demonstrations of X‐ray and multi‐mode sensing from lead‐based colloidal thin‐films. For instance, in 2023, X‐ray, optical, and near‐infrared multi‐mode imaging was demonstrated from a colloidal quantum‐dot pixel array composed of PbS nanoparticles.^[^ ^19^ ^]^ The paper demonstrates that colloidal PbS is more robust to X‐ray flux than single‐crystalline PbS, but for high‐energy X‐ray and γ‐ray spectroscopy, the active layer, at ≈900 nm,^[^ ^19^ ^]^ is too thin because of information loss associated with secondary electron escape. Some thicker X‐ray sensing stacks have been created, such as a 2 µm thick PbS X‐ray monitor^[^ ^20^ ^]^ or thicker hybrid PbS/organic sensing stacks,^[^ ^21^ ^]^ but they haven't been designed nor evaluated for photon spectroscopy. Furthermore, the spin‐cast and layer‐by‐layer methods used to form the X‐ray flux sensors cannot be readily scaled to thicknesses that are 1000 to 10 000 times greater than those needed for flux monitoring.

One strategy by which one can incorporate size‐tunable quantum dots within a thick sensing material is to embed the nanoparticles into a polymer and form an indirect detector, in which scintillation photons created by the impinging quanta are sensed by an adjoining photosensor. The composite nanoscintillators are typically limited by the low mass‐fraction of the embedded semiconducting nanoparticles, resulting in: 1) poor spectroscopic performance due to energy loss in the surrounding organic, and 2) a low photopeak‐to‐total ratio because of dominance of Compton scattering within the filler.^[^ ^22^, ^23^ ^]^

In this paper, we discuss an inherently facile, scalable detection scheme in which the energy of the incident quanta is converted directly into electron‐hole pairs. In terms of the novelty of the synthesis, lead chalcogenide nanoparticles are primarily synthesized within an organic solvent and an autoclave, whether forming binary compound, alloys, or core‐shell (PbTe@PbS) nanoparticles.^[^ ^24^, ^25^, ^26^, ^27^ ^]^ The main drawback of using organic syntheses is that the long‐chained ligands typically employed to functionalize the nanoparticles (NPs) restrict charge transport when one is attempting to fabricate a large‐volume sensor from the colloidal dispersion. Several trials have also been made to synthesize PbTe in water at a lower temperature. Particularly, Liu et al. 2012^[^ ^28^ ^]^ and Wang et al. 2009^[^ ^29^ ^]^ synthesized PbTe in water; however, the morphological properties of the crystals were not well‐defined, with the presence of a mixture of polyhedron, cubic, and spherical structures. In this article, we report on the facile aqueous synthesis of PbS_x_Te_y_ nanoparticles in ambient environments at low temperatures. We report on extensive studies of nanoparticles’ composition, size, and shape modulation.

From a device perspective, the lead chalcogenides are particularly attractive semiconductors because they allow band‐gap design flexibility as their relatively low bulk band‐gaps can be combined with large Bohr radii to allow one to modulate the band‐gap across a range of 0.3–4 eV via careful size and ligand control when formed into nanoscale form. Hammig (2012) and Davis et al. (2021) showed that high‐resolution X‐ray and γ‐ray sensors could be created via the self‐assembly of PbSe NPs from either drop‐casting^[^ ^30^ ^]^ or solution‐based colloidal crystal growth,^[^ ^31^ ^]^ respectively. However, neither method allows one to readily scale the material system to large volumes with form factor flexibility. For spectroscopic X‐ray panels, one desires a fabrication method that allows one to produce a uniform large‐area panels, and for γ‐ray detection, achieving acceptable detection efficiency can require centimeters of detector depth. The outstanding challenge presented by interfacial‐laden materials, such as polycrystalline solids or nanostructured media, is finding the means to efficiently and rapidly surmount the interstitial spaces between the semiconducting crystallites.

Vecchio et al. (2022) showed that one can self‐assemble PbTe NPs into a percolating network with mechanical and electrical properties that were governed by the nanoscale interconnectivity of the solid.^[^ ^32^ ^]^ Although the synthesis methods described can be scaled to large volumes, the open structure of the percolating network results in poor intrinsic detection efficiency. Furthermore, the diameters of the PbTe NPs produced are highly stable at roughly 4 nm, regardless of changes to the recipe used in their formation, which prevents control over the band‐gaps of the constituent NPs, a deficiency that partially motivates the NP recipe development in this paper.

**Figure 1:**
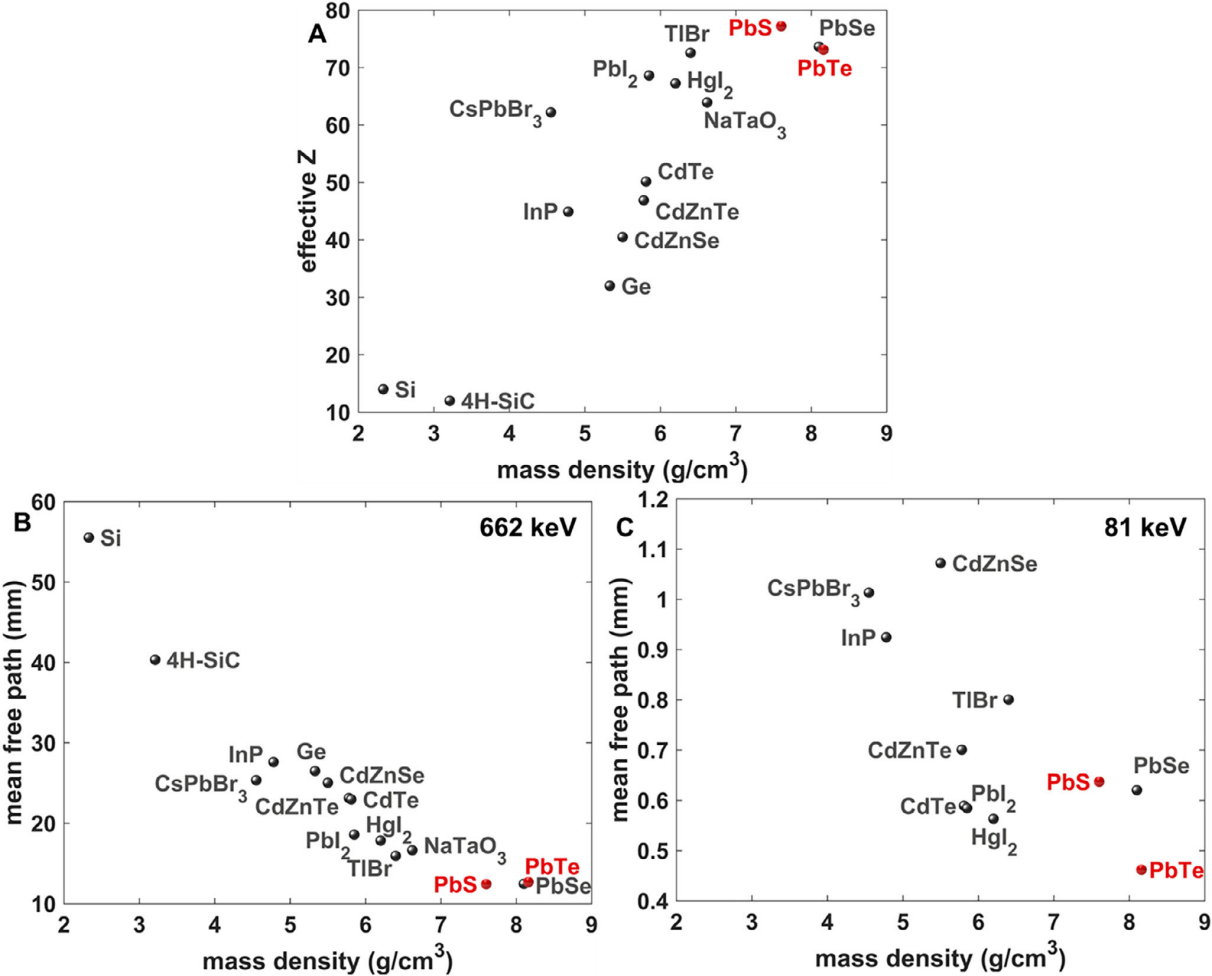
a) *Z*_eff_ and ρ map of various semiconducting materials used for photonic sensing, with the material combinations reported highlighted in red. The effective *Z* is calculated from formula 4.4 as reported by Jackson and Hawkes,^[^ ^37^ ^]^ and the densities are assumed to be the bulk density quoted in the Materials Project Database.^[^ ^38^ ^]^ Note that for nanostructured media, the degree to which the density approaches this bulk value depends on the packing density and nanoparticle geometry. b,c) *λ* and ρ map of various semiconducting materials used for photonic sensing evaluated at: b) 662 keV and c) 81 keV. The materials under study are highlighted in red. For the 81 keV plot, the *λ* for Si (19.5 mm), SiC (15.4 mm) and Ge (2.04 mm) are beyond the range shown.

Vecchio et al. (2025) introduced the use of an aramid nanofiber (ANF) matrix as a scaffold upon which one could graft cadmium telluride (CdTe) NPs to form a high‐resolution sensor.^[^ ^3^ ^]^ The ANF provided a template upon which close‐packing could be achieved for NPs functionalized by short‐chained ligands (e.g. thioglycolic acid), the latter facilitating effective charge‐transport without the need for ligand‐exchange, functionalization, or annealing. This device fabrication approach involves a simple vacuum filtration set‐up where the nanoparticle colloid is fluxed directly through the ANF without the need for any other equipment or chemical processes. Nevertheless, the resolution loss due to statistical counting noise can be limited by the height of the effective band‐gap, and one would prefer to start with a bulk material with a smaller bulk bandgap compared to that of CdTe (1.5 eV). Furthermore, as shown in **Figure** **1**, for X‐ray and γ‐ray detection efficiency, one would prefer to replace the Cd with Pb to take advantage of its higher intrinsic efficiency.

When choosing a material for the active sensing layer, compounds with a high effective atomic number enable efficient thin‐film sensors of X‐rays and γ‐rays. A map of various elemental, binary, and ternary compounds in comparison to the effective Z (*Z_eff_*)^[^ ^33^ ^]^ and density of PbS and PbTe is provided in Figure 1A, the combination of which is the focus of this paper. From a photonic‐stopping perspective, PbS_x_Te_y_ has the highest *achievable* density of the compounds shown, and it has a higher density and *Z_eff_* than the cadmium compounds, such as CdZnTe. The lead chalcogenides (PbS, PbSe, PbTe) have substantially higher *Z_eff_*’s than common sensing medium, but because their band‐gaps in single‐crystalline form are so low (PbS: 0.45 eV, PbTe: 0.30 eV),^[^ ^34^ ^]^ quantum confinement must be exploited to increase the size‐dependent band‐gap to mitigate thermally‐generated leakage current, noting that the band‐gap of germanium, at 0.66 eV,^[^ ^35^ ^]^ necessitates cooling to liquid nitrogen temperatures (77 K) to quench this noise. The lead chalcogenides possess high dielectric constants and small effective masses, the consequence of which is that their exciton Bohr radii are larger than those of the cadmium chalcogenides. For instance, the Bohr radius of CdSe is 6 nm while that of PbSe is 66 nm.^[^ ^24^ ^]^ Thus, relative to the cadmium compounds, colloidal solids comprising larger nanoparticles can be used to modulate the band‐gap of the semiconductor.

Using the total microscopic cross‐section derived from the XCOM database,^[^ ^36^ ^]^ the mean free paths (l) of 662 keV γ‐rays from ^137^Cs and 81 keV γ‐rays from ^133^Ba are shown in Figure 1B,C, respectively, for the various semiconductors shown in Figure 1A. In consonance with the *Z_eff_* plot, the lead chalcogenides have a lower l than the other common bulk semiconductors used for γ‐ray and X‐ray detection (HPGe, Si, CdTe, CdZnT, CsPbBr_3_), and they therefore serve as a promising material family for photonic sensing media. However, because they must be nanostructured to achieve spectroscopic detection of single photon interactions, one requires synthesis methods that produce effective charge percolation across large macroscopic volumes with nanoscale ordering.

The work described here shows that one can successfully integrate PbS_x_Te_y_ nanoscale clusters within an ANF matrix to make an effective X‐ray and γ‐ray sensor. In order to achieve better charge‐conversion efficiency, this report discusses an aqueous synthesis approach that allows one to vary the band‐gap via the formation of PbS_x_Te_y_ via size and compositional control. Those NPs can be effectively integrated into various thicknesses of ANF so that scalability in area and depth is achieved.

Regarding sensor fabrication techniques, there have recently also been multiple advances in making thick X‐ray and/or γ ray sensors by different techniques such as electrohydrodynamic jet‐printed, solution growth, spray‐coating, doctor‐blade coating, hot spin‐coating, or inkjet‐printed. Tortora et al. fabricated iodide‐exchanged PbS quantum dots X‐ray sensors using electrohydrodynamic jet (EHD‐Jet) printing. With silicon substrates and interdigitated gold contacts, the authors printed colloidal PbS quantum dots in toluene supported by an applied electric field. With this technique, the authors report consistency and uniformity of the active material, though micrometer‐sized cracks and residual crystallites on the surface are still spotted after the iodide ligand exchange process.^[^ ^39^ ^]^ Similar to this process, other conventional inkjet printing techniques also involve the ejection of semiconducting materials onto a substrate, but rather than being supported by voltage the ink is usually combined with organic compounds for better ink ejection and adhesion, such as diethylene glycol mono methyl ether,^[^ ^40^ ^]^ methylammonium, and formamidinium.^[^ ^41^, ^42^, ^43^, ^44^ ^]^ For coating methods, notably, using roughly the same ink composition with formamidinium iodide precursor, Dang et al. also demonstrated earlier this year that a thicker formamidinium lead halide perovskite device with good X‐ray attenuation can also be fabricated via a simple spin–coating stage where the solution was spin–coated on the electrodes at 60 °C.^[^ ^45^ ^]^ Different coating techniques are also used by different groups during the last 5 years also include spray coating CsPbIBr_2_ with double additives of NH_4_SCN and L‐α‐phosphatidylcholine to improve the coverage rate of substrates and stability and crystallinity of the films^[^ ^46^ ^]^ or fast doctor‐blade coating methylammonium lead bromide with 18‐crown ether‐6 to increase the solution viscosity and film thickness.^[^ ^47^ ^]^ These approaches, despite their success in device sensitivity and apparent scalability, are all subjected to further complex processing as follows: a) coating solution, most of which involve the use of formamidinium with careful ratio selection within the solution and b) even for solution growth approaches,^[^ ^48^, ^49^ ^]^ are subjected to substrate processing to ensure a clean and ready surface for later incorporation of the energy detecting materials. The fact that these technologies involve additional devices such as a spray‐coater, a spin‐coater, a printer, and a voltage supplier also adds to their cost and lengthy device fabrication.

## 2 Results and Discussion

### 2.1 Nanoparticle Synthesis and Morphology

#### 2.1.1 Non‐Classical Colloidal Crystal Growth

**Figure 2:**
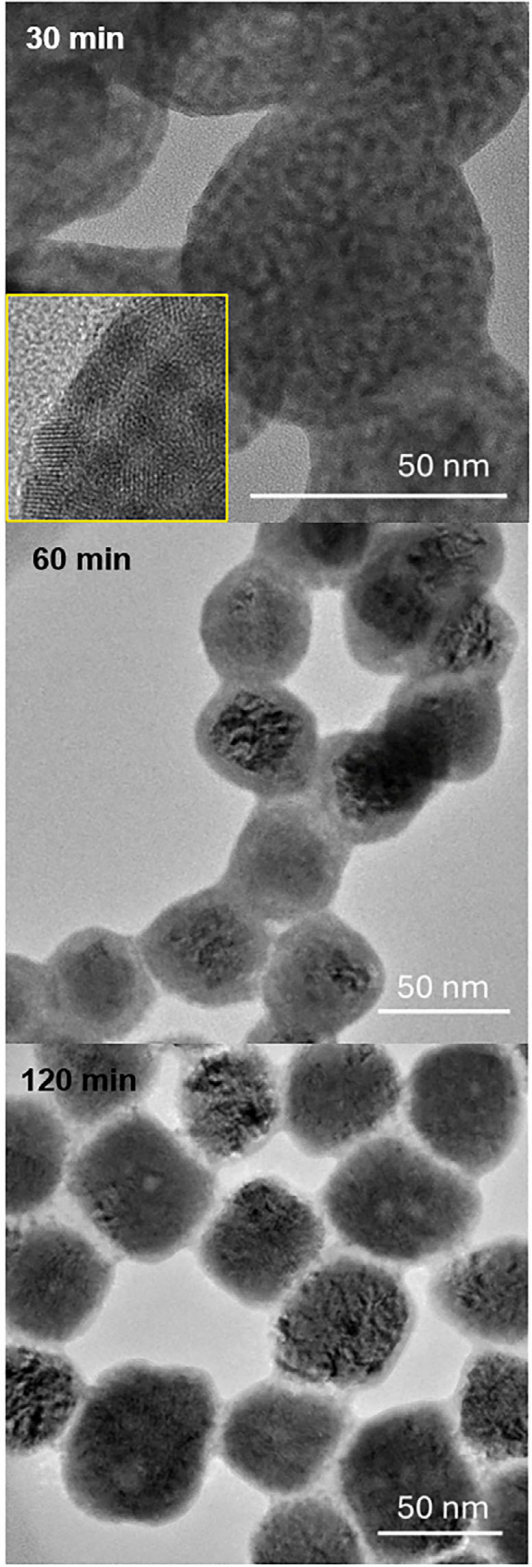
TEM micrographs of PbS_x_Te_y_ NPs for aqueous bath reflux times of 30, 60, and 90 min. The reflux temperature was 100 °C showing the evolution from spherical nanoparticle clusters to cubic colloidal crystallites. The inset picture shows an expanded TEM image of the constituent NPs at a reflux time of 30 min.

In the *Experimental Methods* section, we describe two methods for forming PbS_x_Te_y_ NPs that are each functionalized by thioglycolic acid (TGA). In the first pathway, a single‐ligand (TGA) design is utilized, resulting in a NP size variation that proceeds via a crystallization pathway in which 4–5 nm spherical building blocks are formed into superclusters that limit the extent to which the constituent particles can coalescence, as shown in **Figure** **2**.

The transmission electron microscopy (TEM) micrographs of the colloidal solution show that the NPs undergo non‐traditional crystallization.^[^ ^50^, ^51^ ^]^ Specifically, *atomic* nucleation can be facilitated via the formation of nanoscale clusters or aggregates within which crystalline polymorphs can grow.^[^ ^50^ ^]^ These clusters are thermodynamically stabilized at some critical radius within which the motion of the atoms is constricted, which facilitates bond formation and crystallization.^[^ ^50^, ^51^ ^]^ For the nanoparticle system described in this article, the building blocks are NPs rather than atoms, but a similar mechanism is observed, in which spherical cluster formation in the 30 min micrograph of Figure 2 is followed by (colloidal) crystal growth over the following 90 min. Instead of the atoms adding epitaxially upon any nuclei that are homogenously formed within a colloidal solution (the traditional crystal‐growth path), the NPs instead form a dynamic flexing assemblage (30 min image of Figure 2). Within these aggregates, the 10's to 100's of NPs have enough affinity to form clusters within which the motion of the NPs is restricted to reduce high surface‐energy contributions. If allowed to stay in this pre‐critical nuclear state, then cubic *colloidal* crystals begin to form in the interiors of the pre‐critical clusters. If one looks closely at the 30‐ and 60 min TEM micrographs in Figure 2, a cubic crystal is shown to form within the spherical cluster. The cubic lattice is not a single PbS_x_Te_y_ NP; rather, it is a colloidal crystal composed of ≈4–5 nm PbS_x_Te_y_ NPs, as highlighted in the inset picture of the 30 min panel of Figure 2. While the size of the nanocluster grows, the inset micrograph shows that the cubic NPs are multicrystalline, and these domains of quantum confinement are only slightly enhanced from the initially formed NPs.

#### 2.1.2 Classical Colloidal Crystal Growth via Ostwald Ripening

**Figure 3:**
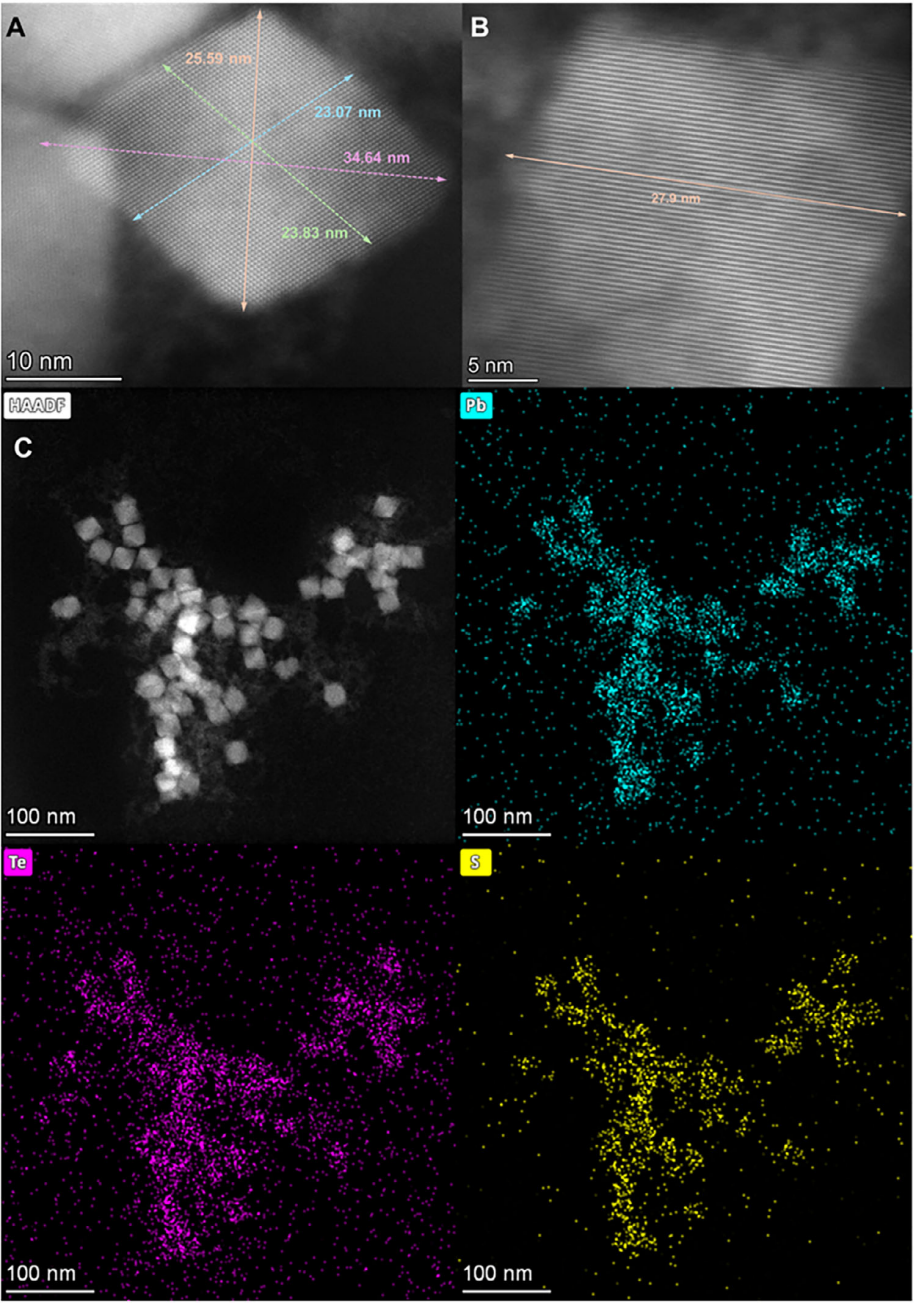
A) STEM micrograph PbS_x_Te_y_ nanocrystallite synthesized at 60 °C using 6.4 mL N_2_H_4_ for 60 min. B) TEM micrograph PbS_x_Te_y_ nanocrystallite synthesized at 60 °C using 6.4 mL N_2_H_4_ for 60 min. C) STEM HAADF image and associated elemental maps for Pb, Te, and S, derived from EDS X‐ray spectrum.

The single‐ligand synthesis approach prevents one from strongly modulating the electronic band‐gap via nanostructural size‐control because the quantum confinement is governed by the cluster's constituent NPs that are fixed at diameters in the 4–5 nm range. In order to realize size control and therefore band‐gap control of the NPs, we added a longer‐chain ligand, polyvinylpyrrolidone (PVP), to the TGA as a means to prevent the formation of the stable superclusters (cf. Experimental Section). SEM imaging can be used to estimate the composition and size variation in the nanoparticles as the process parameters are varied, but the composition and nanostructural geometry can be more precisely gauged using scanning transmission electron microscopy (STEM) and X‐ray diffraction (XRD) spectroscopy. Nanoclusters, like those shown in Figure 2 are frequently observed in other nanoparticle syntheses, especially with gold^[^ ^52^, ^53^ ^]^ or polycrystalline supraparticles. **Figure** **3**A,B shows that the PVP successfully results in the formation of single‐phase nanocrystallites as desired, so that the size of the particle can be used to control the optoelectronic properties of the semiconducting structures.

**Figure 4:**
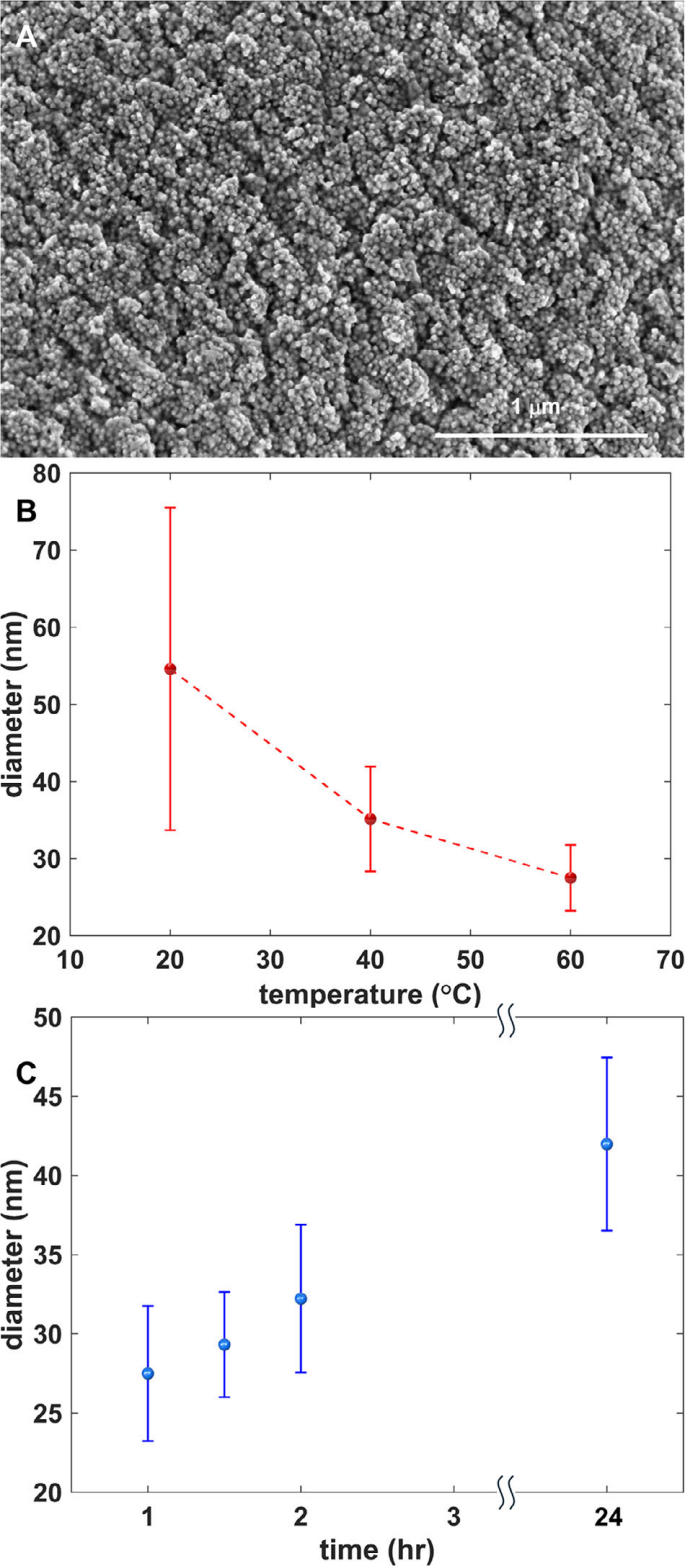
A) SEM micrograph of PbS_x_Te_y_ nanoparticles synthesized with 6.4 mL N_2_H_4_ using NaOH as the base at 60 °C (MOE = ± 2 °C) for 60 min. B) Variation in the nanoparticle size as the reaction temperature is varied (cf. Figures S6–S8, Supporting Information). C) Variation in nanoparticle size as reaction time is varied for the 60 °C bath using 6.4 mL N_2_H_4_ as derived from SEM micrographs (cf. Figures S3–S5, Supporting Information). The diameter data are represented as the mean ± standard deviation of all independent particles measured on the SEM views, which total to 50–100 nanoparticles from 10 to 20 fields per approximation.

As shown in Figure 3A, faceted nanocrystallites are observed that have spatial extents in the 23–30 nm diameter range. Figure 3A shows the clear single‐crystalline lattice of a PbS_x_Te_y_ nanoparticle that is imaged. Noting that there can be foreshortening in the image and therefore the diagonal length is most indicative of the particle size, the side length, at 24.49 nm, is similar to those shown in the SEM images in **Figure** **4** and in Figure S1 (Supporting Information). The measured lattice spacing of the nanoparticles in Figure 3B is shown to be roughly 2.9 Å, which is the Pb─S bond distance in the lattice of PbS.^[^ ^54^ ^]^ The high‐angle annular dark‐field (HAADF) STEM image in Figure 3C also reveals smaller particulates that are not integrated into larger nanocrystallites, which can modify the optoelectronic properties of the overall colloidal solution by producing scattering and absorption centers. The elemental maps of Figure 3C, which are derived from the energy dispersive spectroscopy (EDS) distributions – show that Pd, S, and Te are all localized to the NPs shown in the HAADF image. There are no moiré patterns or concentrations of tellurium on the surfaces, either of which can suggest the formation of PbTe@PbS core@shell NPs,^[^ ^26^ ^]^ suggesting that ternary compounds of lead, sulfur, and tellurium are formed, a conclusion buttressed by XRD analysis of the solids.

Size modulation can be achieved via either temperature or reflux‐time control. To study the effect of solution temperature on the PbS_x_Te_y_ NPs growth within the colloid, the reaction was run for a constant 60 min duration and reacted with a fixed amount of 6.4 mL N_2_H_4_, while the temperature was varied from 20 to 80 °C. Figure 4A shows an example SEM micrograph of the top surface of the composite for NPs grown at 60 °C and 60 min. The size modulation trend is plotted in Figure 4B. All syntheses yield polygonal PbS_x_Te_y_ nanoparticles except for the sample synthesized at 80 °C (Figure S2, Supporting Information). In that case, the top surface of the PbS_x_Te_y_/ANF composite exhibits rod‐like structures of PbS_x_Te_y_ instead of polygonal PbS_x_Te_y_ like all other samples, including the bottom side of the 80 °C sample shown in Figure S2 (Supporting Information). Along with the syntheses of PbS_x_Te_y_ and PbTe nanoparticles,^[^ ^27^ ^]^ the literature has shown that higher temperatures can encourage the growth of PbTe nanowires, especially with the use of PVP.^[^ ^55^ ^]^ The larger extents of the nanorods at 80 °C prevents their ready fluidic transport through the ANF pores. In contrast, the cubic/monoclinic and smaller nanoparticles within the colloidal solution can flow through the pores and bond along the ANF, including along the bottom surface of the scaffold.

As shown in Figure 4B, there is a steady decline in the average diameter of the nanoparticles when the reaction temperature is increased. On the basis of Lamer's Burst Nucleation mechanism,^[^ ^56^ ^]^ an increased temperature improves the uniformity of the nucleation during precursor injection because of enhanced component‐mixing, the effect of which is a greater number of nuclei formed for NP growth, from which a smaller ultimate particle size results. What is more, it can also be seen clearly from Figure 4B that the standard deviation in the average diameter decreases consistently when the reaction temperature is increased via the same mechanism.

To study the impact of the reaction time on NPs growth, the reaction temperature is held constant at 60 °C for all syntheses and a constant amount of 6.4 mL of N_2_H_4_ is used. The reaction time is then varied as follows: 1, 1.5, 2, and 24 h. As the reaction time increases, the particle diameter increases, as reflected in the summary plot of Figure 4C, derived from SEM images Figures S3–S5 (Supporting Information). The Ostwald ripening effect^[^ ^57^, ^58^ ^]^ is due to the differences in the solubility of nanoparticles with different sizes, where smaller nanoparticles with higher surface energy dissolve to reduce the total energy of the solution. The NP size doesn't change appreciably when the reducing agent concentration is changed (Figures S9–S11, Supporting Information).

### 2.2 Structural and Optical Properties

**Figure 5:**
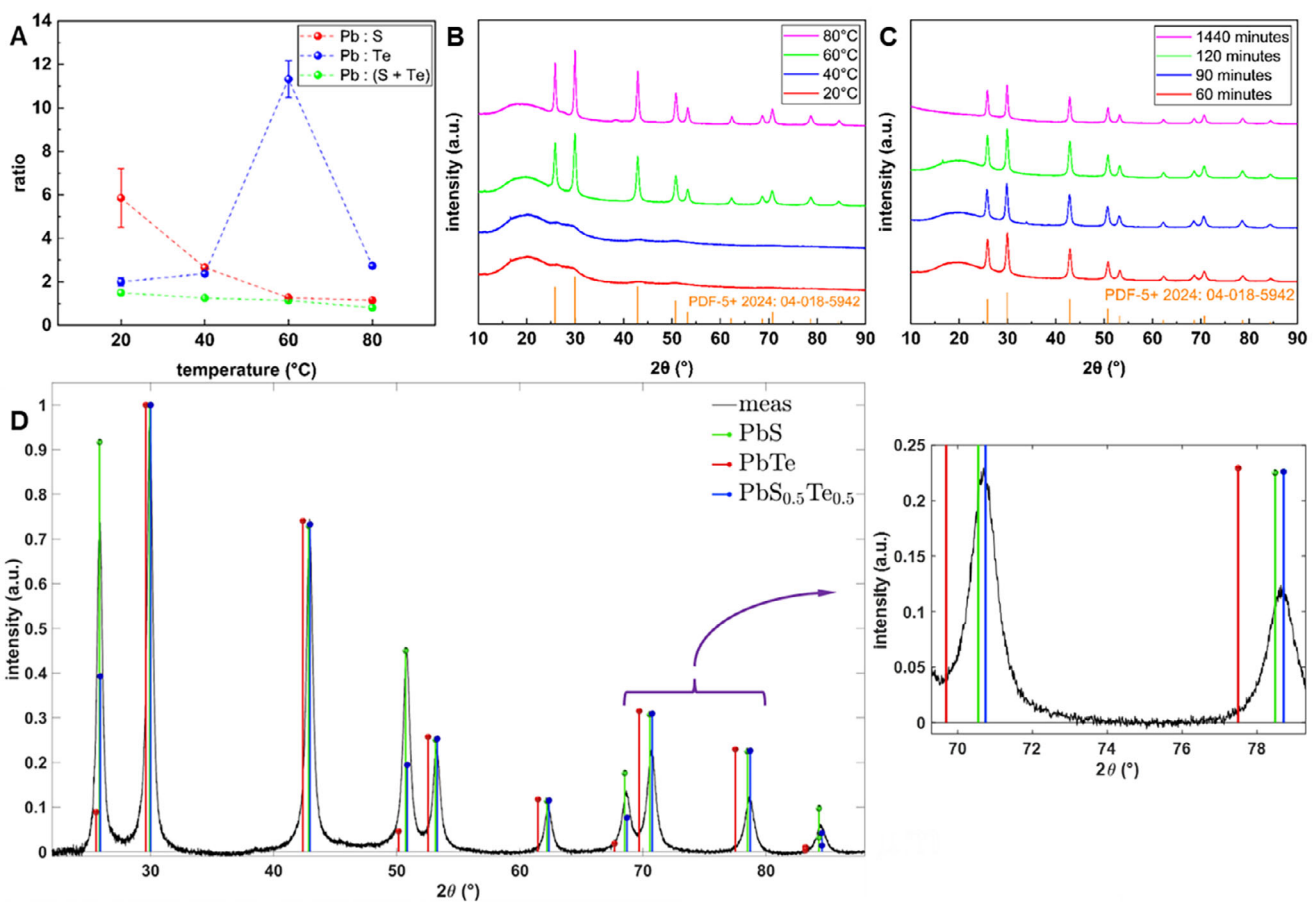
A) On an atomic basis, the ratios of the elements identified in the legend as derived from SEM/EDS spectroscopy for the various water bath temperatures. B) XRD analysis for PbS_x_Te_y_ synthesized using 6.4 mL N_2_H_4_ (100x) at the temperatures: 20, 40, 60, 80 °C for 60 min. (C) XRD spectra for 60, 90, 120, and 1440 min (24 h) at 60 °C. The broad hump between 15 and 35 °C is due to the ANF scaffold. The orange library file is the powder XRD spectrum for PbS_0.9_Te_0.1_ compound. D) After subtraction of the ANF background, the measured XRD spectra (for 60 °C, 100:1 N_2_H_4_ synthesis) compared to library files for PbS (04‐004‐5639), PbTe (04‐016‐9844), and PbS_0.5_Te_0.5_ (04‐023‐6550). The inset shows the expanded view between 69 and 79 degrees so that the superior match in the ternary compound can be clearly seen.

The successful synthesis of PbS_x_Te_y_ is supported by the SEM‐derived energy‐dispersive X‐ray spectroscopic (EDS) analysis of the material. The X‐ray lines M_α_, M_β_, M_γ_ of Pb, K_α_ of S, and L_α_, L_β_ of Te all appear on the spectra. Oxygen and carbon peaks are also observed in the EDS spectra, which are due to the organic capping ligands and the polyamide chain, which is the building block of the ANF. **Figure** **5**A is a plot for the Pb to S atomic ratio, the Pb to Te ratio, and Pb to (S + Te) ratio of all PbS_x_Te_y_ nanocrystallite samples whose reaction temperatures are varied. The spectra and quantification tables for this variation are included as Tables S2–S5 and Figures S12–S15 in the *Supporting Information*. One can observe from the green trace in Figure 5A that for all samples, the Pb concentration is roughly equal to the sum of the atomic concentrations of S and Te, regardless of temperature. Generally, the S atomic percentage increases with the reaction temperature, whereas the Te contribution decreases. The data also indicates that the sample synthesized at 80 °C is the only material where the S and Te combined concentration is larger than the atomic concentration of Pb, which can reflect the formation of Te‐nanorods in addition to the cubic PbS_x_Te_y_ nanoparticles. Further XRD analysis is performed to identify crystalline phases in the material, but based on SEM EDS spectra, the molecular formulae for samples synthesized at 20, 40, 60, and 80 °C are estimated to be PbS_0.25_Te_0.75_, PbS_0.5_Te_0.5_, PbS_0.9_Te_0.1_, and PbS_0.7_Te_0.3_.

As can be seen from Figure 5B,D, measured XRD spectra contain diffraction signals that can be attributed to the lead sulfide telluride (PbS_x_Te_y_) structure. These indicative peaks are at 25.88° (1 1 1), 29.97° (2 0 0), 42.90° (2 2 0), 50.78° (3 1 1), 53.21° (2 2 2), 62.28° (4 0 0), 68.60 (3 3 1), 70.65 (4 2 0), 78.60 (4 2 2), 84.41 (5 1 1/3 3 3). For samples synthesized at 20 and 40 °C (cf. Figure 5B), although there are broad features near the expected peak positions, the poorer crystallinity and lower NP loading within the composite result in a broadened and barely discernible peak. Aside from these samples, all other XRD graphs are well‐defined with similar peak intensities and widths. This aligns with the average diameter calculations summarized in Figure 4, which shows that only a weak size‐modulation is present for the parameters that are varied. Only two XRD analysis spectra: 1) temperature variation, and 2) time variation runs are shown here in Figure 5B,C; the XRD analysis spectra of the reducing agent concentration variation are included in the Supporting Information section as Figure S16 (Supporting Information).

Figure 5D shows the XRD spectra for the standard synthesis procedure (60 °C, 100x N_2_H_4_) compared with library files from PbS, PbTe, and PbS_0.5_Te_0.5_. The PbTe library file doesn't match the observed distribution, but the observed spectrum shows a gradual, steady increase in the *2θ* separation compared to the PbS library, and measured data indicate a compositional difference between the measured data and either pure PbS or core@shell PbTe@PbS. As shown in the blue stem plot, the composition matches that of ternary PbS_x_Te_y_ compounds with sulfur concentration between 0.4 and 0.6, with 0.5 utilized in the figure. This is clarified in the expanded view in the inset picture for the larger values of *2θ*. The lattice constant, as derived from the spectra, is 0.5960 nm, a value between that reported for PbS (0.5935 nm) and PbTe (0.6439 nm).^[^ ^59^ ^]^

**Figure 6:**
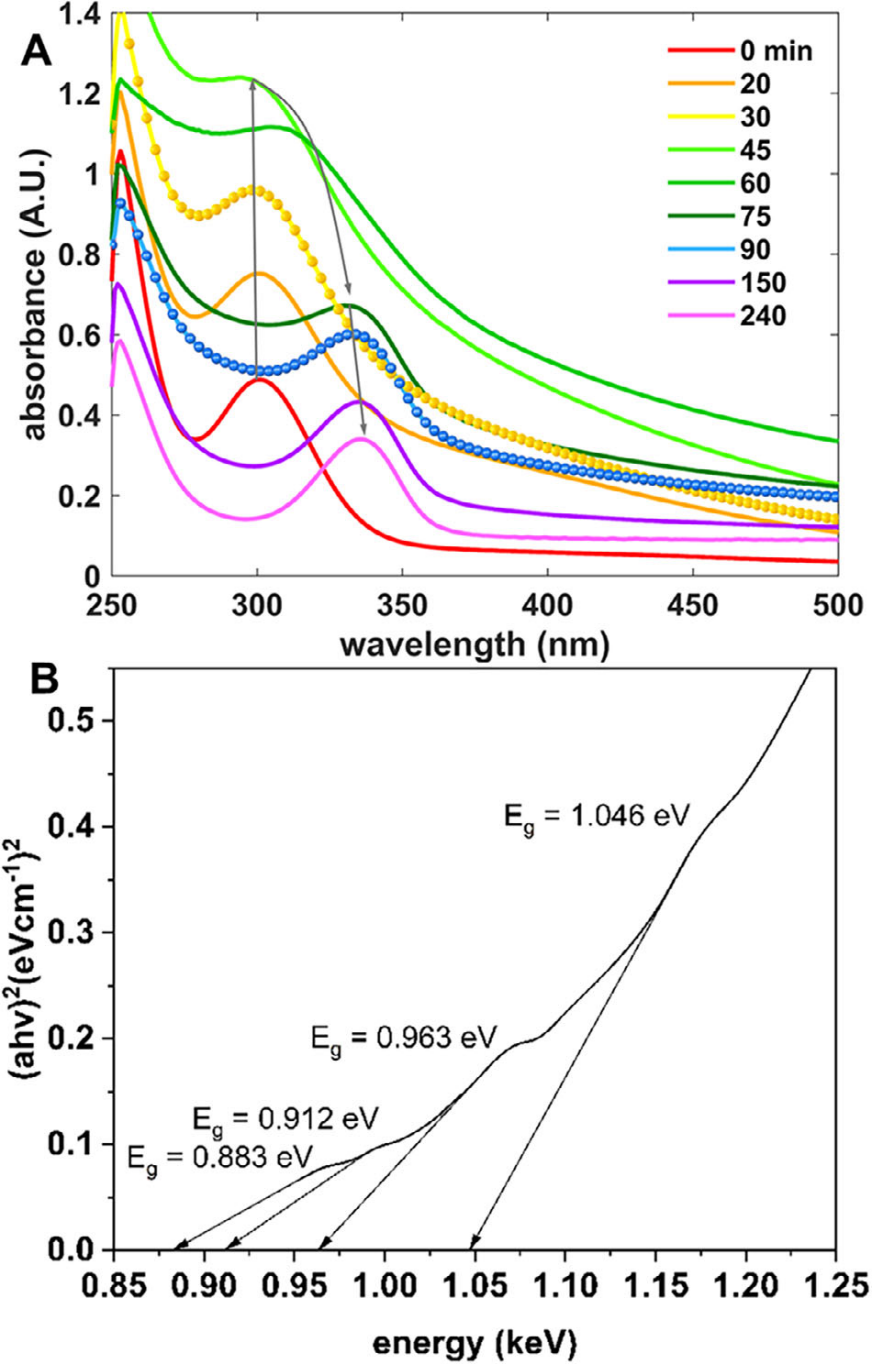
A) Absorbance spectra of the PbS_x_Te_y_ nanoclusters colloidal solution as a function of time. The grey arrow shows the temporal path of the absorbance. The two curves displayed with (yellow and blue) spherical markers highlight the 30 to 90 min morphological transition as cubic colloidal crystal formation is realized within the superclusters (cf. Figure 2). B) As derived from the UV–vis–NIR absorbance spectrum of PbS_x_Te_y_ nanoparticles synthesized at 60 °C using 6.4 mL N_2_H_4_ (100x) for 60 min (cf. Figure S17, Supporting Information), the Tauc plot as a function of energy.

For sensing applications, the band‐gap is used to modulate both the amplitude of the thermally generated noise and the size and stochasticity of the signal. For the TGA synthesis that produces PbS_x_Te_y_ nanoclusters comprising 4–5 nm constituent NPs, the band‐gap, at ≈3.4 eV, can be discerned from the projection of the absorbance edge of the right‐most peak (336 nm) of **Figure** **6**A. In contrast to Ostwald ripening, in which a steady red shift of the absorbance peak can reflect the growth of the overall NP distribution, the peak at 300 nm abruptly red‐shifts between 30 and 90 min, as highlighted in the demarked yellow and blue curves in the plot. The lowering of the band‐gap is due to relaxation in the strong‐confinement due to the longer‐range bonding order of the NPs, as shown in the close‐packed cubic bonding of the NPs shown in the inset picture of Figure 2. For higher intrinsic sensor resolution, one desires a small band‐gap to minimize the statistical counting noise associated with discretizing the energy into electron‐hole pairs. The degree to which one can reduce the band‐gap is limited by thermal carrier generation, but that noise can be quenched via the phonon bottleneck effect.^[^ ^3^, ^30^ ^]^

The lack of band‐gap control in the nanocluster colloid motivated the chemical synthesis path that includes PVP. Figure S17 (Supporting Information) shows that the UV–vis absorption is broadband across the optical range up to at least 1300 nm. There are small absorption steps near 600 and 1200 nm, the latter corresponding to band‐gaps near 1 eV, as shown in Figure 6B Tauc plot,^[^ ^60^, ^61^ ^]^ but no strong single exciton peak is observed for the colloid. For the relatively large nanoparticles used in this study, quantization effects are not observed, and therefore, the Tauc plot is appropriate.^[^ ^62^ ^]^ A band‐gap elevated to the 1 eV range is also supported by the limited leakage current of the devices under bias; for instance, when the device used to derive the spectral results of Figure 11 is biased to 143.9 V, the measured leakage current is 2.7 nA.

### 2.3 Composite Film Formation and Device Fabrication

**Figure 7:**
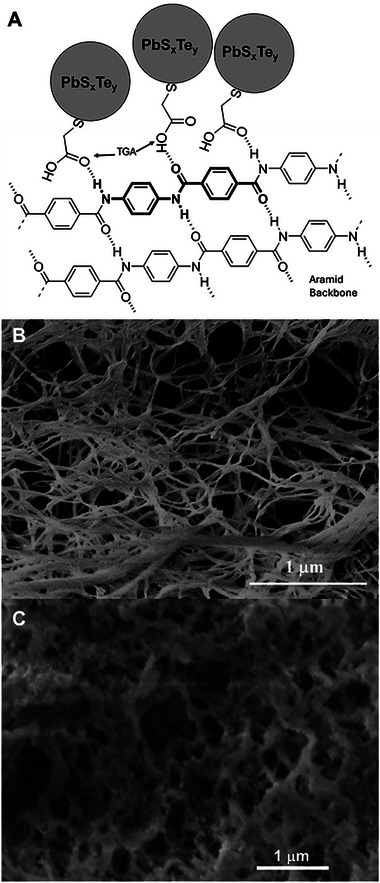
A) Schematic diagram of PbS_x_Te_y_ NP bonding upon the ANF. B,C) Cross‐sectional SEM images of mold‐cast ANF samples before B) and after C) incorporation of PbS_x_Te_y_ nanoparticles.

Whether composed of nanoclusters or nanocrystallites, the colloidal solutions can be flowed through a hydrogel comprising ANFs and functionalized with ligands that can hydrogen‐bond upon the amide groups, as shown schematically in **Figure** **7**A. The mass loading of the composite is first defined qualitatively by the visible amounts of PbS_x_Te_y_ nanoparticles that are effectively bonded onto the ANF structure. For instance, Figure 7B,C show SEM micrographs of the mold‐cast solid both without (Figure 7B) and with (Figure 7C) the NP infiltration. Clear PbS_x_Te_y_ NPs can be found bonding onto the fibers as revealed via the encrustation of the fibers compared to the bulk structure of ANF when no particles are introduced. Further surface and cross‐sectional SEM imaging of these samples is included in the Figures S18 and S19 (Supporting Information).

**Figure 8:**
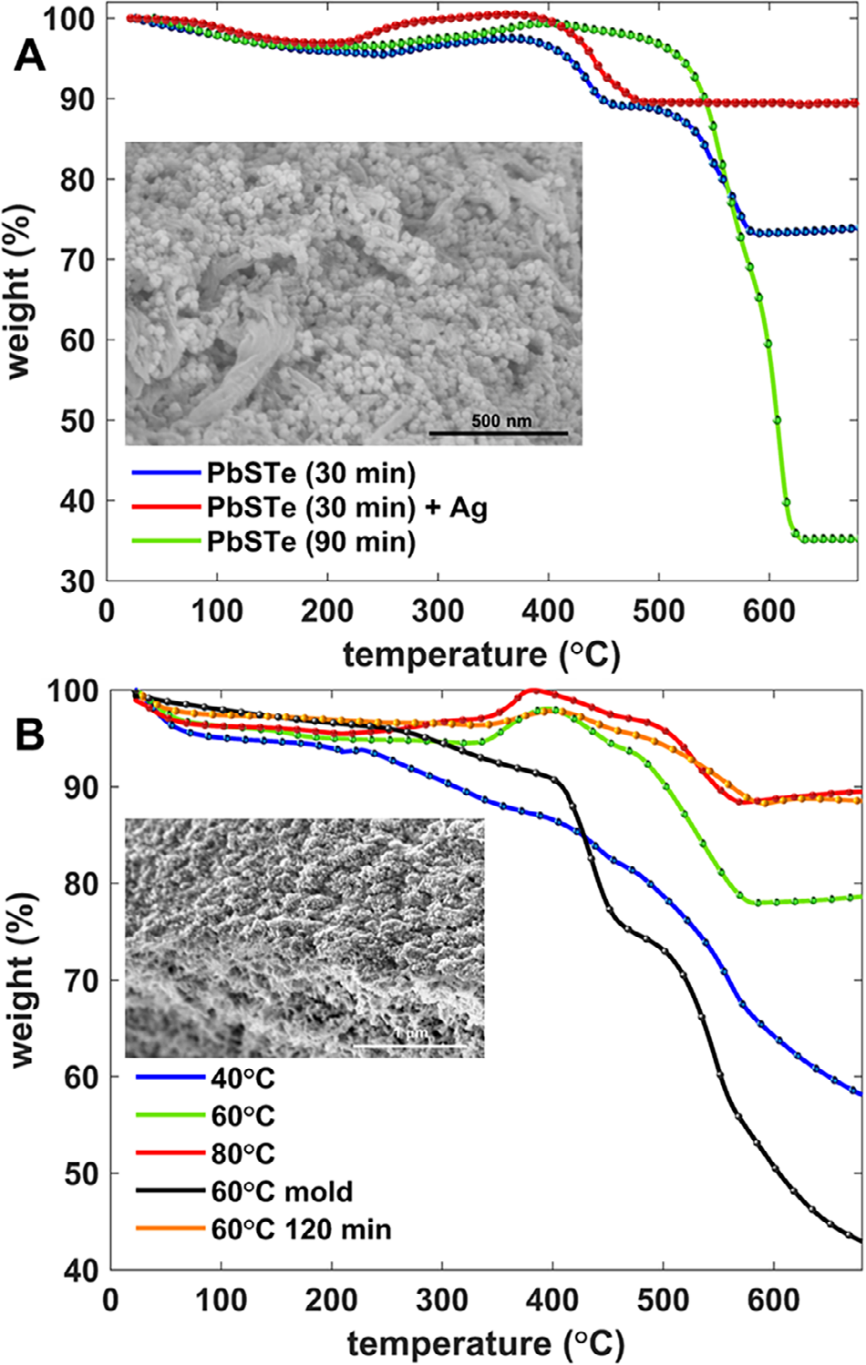
A) Thermogravimetric analysis of PbS_x_Te_y_/ANF derived from TGA solutions with different reflux times (at 100 °C) as indicated in the legend. The inset shows a SEM micrograph of the surface of a PbS_x_Te_y_/ANF composite. B) Thermogravimetric analysis for PbS_x_Te_y_ synthesized with both TGA and PVP using 6.4 mL N_2_H_4_ (100x) at various reaction temperatures and times. All samples are from spin‐cast ANF unless noted (“mold”), and all are refluxed for 60 min unless noted (“120 min”). Note that the thermogravimetric analysis for the sample that is synthesized at 40 °C has a “noise” at around 200 °C, which is likely due to the vibration of the weigh pan during measurement. The inset shows the surface of the spin‐cast sample for the 60 °C sample.

Quantitatively, the loading capacity of each PbS_x_Te_y_/ANF composite is investigated using thermogravimetric analysis. Above 600 °C, all of the organic building blocks of the ANF will already have decomposed^[^ ^63^ ^]^ and the remaining weight is from the PbS_x_Te_y_ nanoparticles. The sizes of the nanostructures affect the mass loading within the composite. The PbS_x_Te_y_ nanoclusters derived from the non‐traditional crystallization path are small enough relative to the ANF pore size that high solid loading is possible, as shown in the thermogravimetric analysis graph for 30 min in **Figure** **8**A (blue curve). Furthermore, if one adds metallic NPs to the composite to facilitate better charge collection, such as the silver (Ag) NPs described in the Experimental Section, then high mass loading values (88 wt.%) can be observed, as shown in the red curve of Figure 8A. As the reflux time is increased to 90 min and beyond, the outermost NPs are consumed and incorporated into the cubic supercluster, as shown in Figure 2, for the 60 and 120 min time marks. These superclusters can become heavy enough that they fall out of solution, which can ultimately make incorporation lower and further, it can clog the ANF pores (green curve of Figure 8A).

Similarly, for the PbS_x_Te_y_ nanocrystallites, the temperature and time variation samples are tested for the thin film samples as well as with one thick 60 °C PbS_x_Te_y_/ANF composite, with thermogravimetric analyses summarized in Figure 8B. The sample synthesized at 20 °C is tested but not included in the figure due to instrument vibrational noise that could impede the illustration of other graphs. This thermogravimetric analysis is, as a result, included as part of the Supporting Information section (Figure S20, Supporting Information). As can be seen from the thermogravimetric analysis graph, the PbS_x_Te_y_/ANF samples all show two significant mass‐loss transitions at around 400 and 500 °C (aside from the typical water vaporization gradual weight loss from RT to 100 °C). Among those two weight drops, the sharp loss at 500 °C has been observed in a previous paper that worked with ANF, which reported that at this temperature, the polyamide starts to decompose.^[^ ^63^ ^]^ The less intense weight loss at 400 °C is likely to be from the decomposition of the stabilizing ligand, PVP, which starts to decompose at 380 °C.^[^ ^64^, ^65^ ^]^ Note that in several of the graphs, just below the decomposition temperature, the weight percentages can increase, which we attribute to center‐of‐mass shifts of the solid within the balance as the PVP starts to melt and evaporate.

Figure 8B shows that the samples synthesized at lower temperatures, where the NPs are comparable in size to the ANF pores, had poorer NP loading than the samples derived from NPs reacted at higher temperatures. The smaller size NPs with lower size variability results in improved loading because the uniformity of the bonding in depth is facilitated by the continued flow of the NPs through the ANF scaffold as NPs are grafted upon the ANF matrix. At 20 and 40 °C, the large size and non‐uniform shape can result in clogging of the pores in the upper part of the ANF, resulting in pore incorporation and lower loading.

For the 60 and 80 °C samples, for which the NPs are ≈25–30 nm in diameter, the loading is high, at either 78 wt.% (green trace) or 88 wt.% (red trace), respectively (measured at 600 °C). The latter increase in mass loading is also due to the increased mass of the nanorod building blocks that infiltrate the top‐section of the cross‐section. The slight increase in size that accompanies a greater reflux time at 60 °C from 60 to 120 min also results in high mass loading near 90 wt.% (orange trace), whereas refluxing for 1‐day results in particles that are too large to penetrate and incorporate into the ANF.

For the thick mold‐cast ANF, the black trace in Figure 8B shows that mass loading is roughly 52 wt.% after the ANF is decomposed. This lower loading is attributed to a larger scaffold thickness with an identical NP recipe, so that an equivalent number of PbS_x_Te_y_ NPs are spread across a larger volume. Additional flows of more NPs through the mold‐cast ANF did not result in higher mass loading because the NPs are designed, via control of the solution pH, to bond to the ANF but not to each other. This chemical selectivity allows one to scale the synthesis in depth and form thicker solids without clogging, which can potentially facilitate higher detection efficiencies for penetrating quanta.

**Figure 9:**
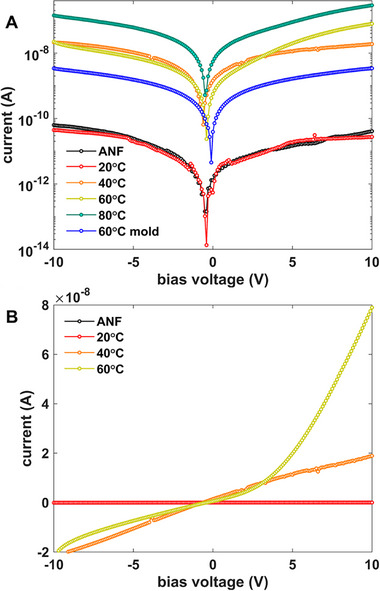
A,B) Current–voltage characteristic of PbS_x_Te_y_ nanoparticle/ANF composite (NP growth temperature in legend) bounded by Cu and Au evaporated electrodes, in semilogarithmic A) and linear B) modes. All are spin‐cast except the noted mold‐cast sample.

The most important function of the ANF scaffold is to provide regularized bonding sites that induce the close‐packing of the semiconducting NPs into a volume‐spanning charge‐percolating network. Regardless of the preparation approach, the semiconductor‐polymeric active layer is bound by evaporated gold and copper contacts to facilitate charge collection (cf. Figure 12 in Experimental Section). The ability of this percolating network geometry to transport charge across the solid depends on the volumetric loading, as reflected in the current–voltage (I–V) characteristics of **Figure** **9**A,B. When the PbS_x_Te_y_ nanocrystallite colloid that was refluxed at 20 °C is flowed through the ANF film, the resulting insulatingI–Vcurve (in red) is similar to that of the bare ANF (in black) because the non‐uniform loading did not result in a percolation path through the matrix. The curves exhibit a zero‐crossing voltage that is slightly below 0 V, reflecting some charge trapping in the sample as the voltage is swept from negative to positive. When swept from positive to negative, a slight positive offset is produced. As the loading increases, the dark current increases. At sufficiently high loading, the I–V curve deviates from a linear ohmic response to a higher slope region, as highlighted in the 60 °C curve (78 wt.% loaded sample) of Figure 9B, which typically reflects contributions from trap‐filled transport.^[^ ^66^ ^]^

### 2.4 Radiation Sensing Performance

The degree to which the flexible composite can transduce photonic interactions into current pulses depends on both the band‐gap and the degree to which NP‐to‐NP interconnectivity spans across volumes large enough to stop the initial neutral quanta, as well as the secondary electrons that result from the interaction. The main function of the ANF scaffold is to enable large‐volume percolation because the strength of the ANF fibers and their bonded coupling to semiconducting nanoparticles result in a macroscopic solid that facilitates multiple avenues for charge transport, enabling continued performance despite any cracks or boundaries that may occur. However, the size of thermally‐generated leakage current impacts the precision of the signal extraction, a value highly dependent on the size‐ and composition‐dependent band‐gap. The supercluster formation shown in Figure 2 results in well‐bonded ≈4 nm PbS_x_Te_y_ NPs with a high enough band‐gap (≈3.5 eV) that the leakage current is negligible. For effective stopping of the incident quanta and secondary electrons, we utilized mold‐cast ANF scaffolds derived from 1 wt.% ANF poured into either 1.5 or 5 mm thick cylindrical molds, noting that during drying, although the lateral dimensions are largely preserved (≈20% diameter reduction), the porous hydrogel shrinks to a final thickness that is roughly 1/10th of the starting thickness because aqueous surface‐tension forces collapse the open network (cf. Experimental Section).

**Figure 10:**
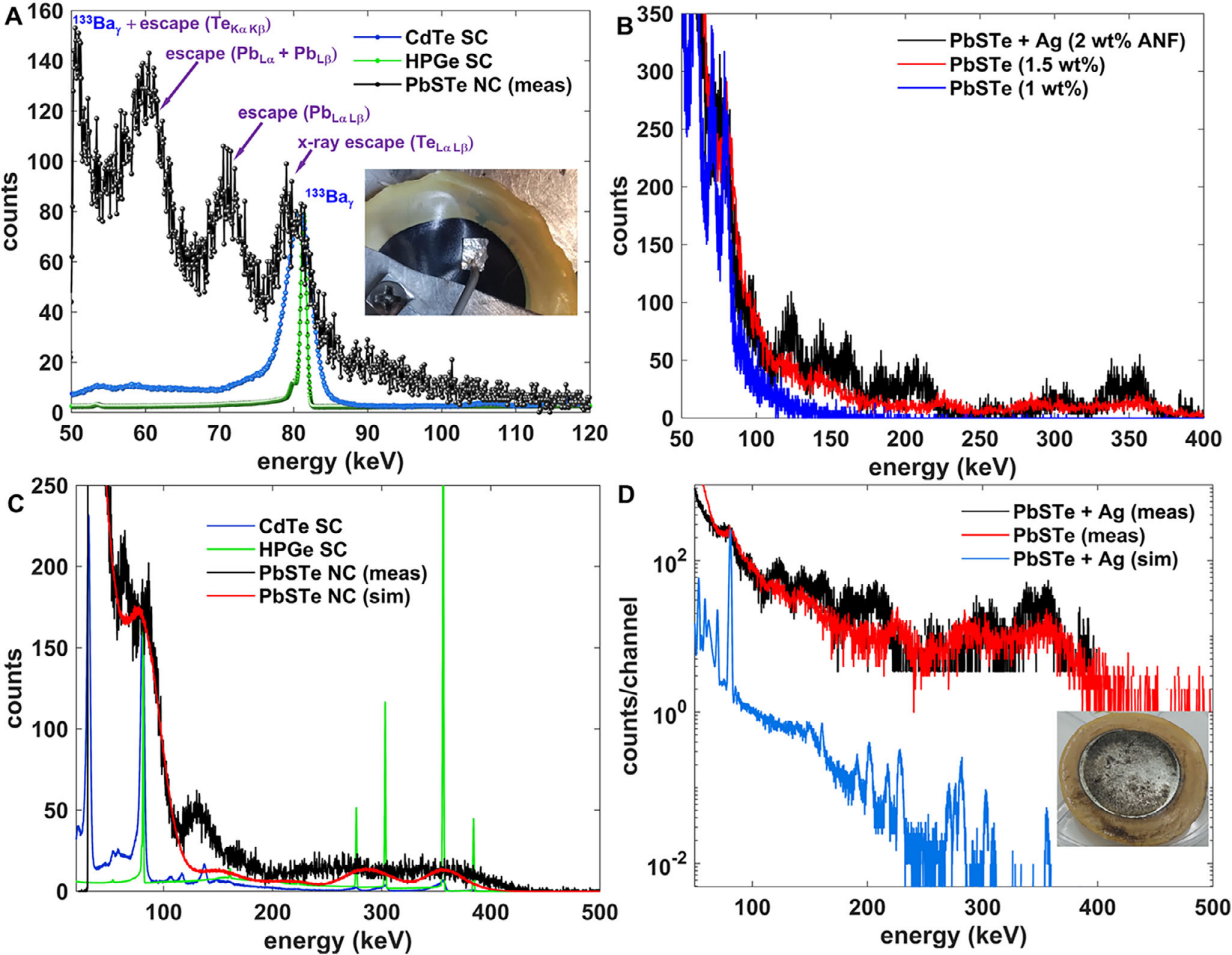
A) Spectral comparison between commercial (1 mm thick from Acrorad) CdTe single‐crystalline (SC) detector (blue), commercial high‐purity germanium (HPGe) in green (3” × 3” liquid‐nitrogen cooled HPGe from Ortec with optimized operating conditions), and nanostructured supraparticle PbS_x_Te_y_/ANF composite, when exposed to X‐rays and γ‐rays emitted from ^133^Ba. In this figure, the legend annotation “PbSTe NC” referred to the nanocrystalline detector composed of supraparticles, not nanocrystallites. The free‐standing PbS_x_Te_y_/ANF solid is shown in the inset. The CdTe Acrorad detector is fully biased (to 700 V) and depleted. The PbS_x_Te_y_/ANF detector is biased to 158.7 V (leakage current < 0.1 nA). Reference X‐ray energies^[^ ^67^ ^]^ are subtracted from the photopeak to identify the source. B) ^133^Ba spectra measured from: (black) PbSTe:Ag/ANF 5mm mold cast solid with 2 wt.% ANF, biased to 144.8 V (leakage current 4.0 nA), (red) PbSTe /ANF 5 mm mold cast 1.5 wt.% ANF sample, biased to 301.0 V (leakage < 0.1 nA), (blue) Expansion of black spectra from (A). The positions of various spectral features are annotated. C) For a spin cast (325 rpm, 2 wt.% ANF) PbS_x_Te_y_/ANF composite contacted with ≈1 cm^2^ Cu foil, the ^133^Ba spectra (in black) compared to that from single‐crystalline CdTe (blue), HPGe (green), and MCNP simulation (red) result. The PbS_x_Te_y_/ANF nanocrystalline composite is biased to 399.9 V (leakage current: 3.7 nA), and the collection time is 15 min. D) On a semi‐logarithmic scale, the comparison between a PbSTe/ANF, PbSTe:Ag/ANF measured ^133^Ba γ‐ray spectra and MCNP‐simulated spectra from PbSTe:Ag/ANF. Photograph of the solid used for the PbSTe + Ag measurement is shown in inset.

Consider the case in which the composition is close to PbS_0.8_Te_0.6_, a composition discerned from SEM‐based Energy Dispersive X‐ray Spectroscopy (EDS) (Table S1, Supporting Information). When a source of X‐rays and γ‐rays (the radioactive isotope ^133^Ba) is exposed to the sample, **Figure** **10**A shows that PbS_0.8_Te_0.6_ solids can achieve excellent energy resolution even for the highly penetrating quanta. Specifically, for the 5.08 cm diameter semicircular section of the 94 µm thick PbS_0.8_Te_0.6_ solid shown in the inset picture, the energy resolution (trace in black) of 2.8 keV (3.5% at 81 keV) is similar to that of a commercial cadmium telluride (CdTe) detector (blue line), but below that derived from a high‐purity germanium (HPGe) detector, shown in the green line, noting that the photopeak width at 81 keV is widened by X‐ray escape features similar to that of the PbSe colloidal solid sensor reported in 2021.^[^ ^31^ ^]^

A consistent feature of our nanostructured composite is that the active semiconducting nanoparticles are bound by organic ligands and intertwined with an organic, insulating scaffold, the consequence of which is that information loss can occur from those X‐rays that emerge from Pb, S, or Te atoms and are subsequently absorbed in the ANF. The X‐ray escape peak structure is therefore typically much more extensive than that of a single‐crystalline equivalent, as indicated in both Figure 10A,B, because the X‐ray doesn't have to escape the macroscopic bounding surface of the device to be lost. Specifically, the 81keV X‐ray escape peaks from the Te L_α_ (3.769 keV) and L_β1_ (4.030 keV) X‐rays, at 77.2 and 76.7 keV, respectively^[^ ^67^ ^]^ overlap just below the 81 keV photopeak in Figure 3A. The Pb L_α_ (10.551 keV) and L_β_ (12.614 keV) escape peaks form a composite feature at 70.4 and 68.4 keV, respectively. Finally, a double escape feature near 60 keV (predicted value 59.9 keV^[^ ^67^ ^]^) is evident.

Figure 10B includes larger volume detectors as well as detectors that include silver (Ag) NPs. Conductive nanoparticles are added after the ANF fibers are encrusted with semiconducting NPs as a means to achieve three objectives: 1) improve the charge transport by bridging any gaps that exist within the percolating network, 2) donate free electrons to fill PbS_x_Te_y_ NP surface traps as a means to passivate the NPs, and 3) add another high‐Z filler to improve the photonic stopping characteristics.^[^ ^3^ ^]^ The two main costs of adding the conductor are that first, the leakage current can increase because of improved percolation, and second, there are more inactive components within which X‐ray interactions can escape. As shown in the caption, the leakage current of the Ag‐included solid increases from less than 0.1 to 4.0 nA, but the resolution with which the spectral information is transduced is higher. Upon comparing the black with the red trace in Figure 10B, the positions of the γ‐ray photopeaks, associated X‐ray escape peaks, and Compton edges are more clearly mapped, the precise labeling included in Figure S21 (Supporting Information).

Figure 10B also shows that the concentration of the ANF dispersion affects the density of the hydrogel scaffold, which then impacts the detection efficiency for incident photons. The concentrations are varied from 1 to 2 wt.%, resulting in a transition from a low‐density solid (blue curve) to a thicker more heavily loaded solid, resulting in effective γ‐ray detection for even the ^133^Ba γ‐rays near 356 keV. As clarified in the spectral comparisons of Figure 10C, the resolution degrades for larger area detectors because the ratio of radiation signal to electronic‐noise degrades. The increased detector capacitance diminishes the size of the voltage pulse from the front‐end charge‐sensitive preamplifier (which integrates the induced current across a combination of the feedback and detector capacitances). However, the detectors achieve the goal of increasing the detection efficiency for the impinging quanta, as evinced in the higher energy spectral mapping as well as the much faster collection times (15 min for red curve, 1 h for black curve vs multiple days for blue curve).

Interestingly, the ability of the detector to fully capture the radiation at high energies (>200 keV) is not expected if one makes a homogeneous solid with the equivalent composition, thickness, and density. One can simulate the distribution of radiation‐induced energy depositions within the homogenous solid with the Monte Carlo N‐Particle Transport Code (MCNP)^[^ ^33^ ^]^ and shown in the blue trace of Figure 10D. The simulated count rate at 356 keV is over three orders of magnitude smaller than that produced by the observed sensor. The main physical reason for the difference is that in the model, the secondary electrons do not stop within the 94 µm thickness of the homogeneous equivalent. As we showed in 2025, via models and measurements, the effect of nanostructuring the solid is to increase scattering within the solid, which results in a more circuitous route of any charged‐particle products.^[^ ^3^ ^]^ The longer residence time is therefore accompanied by greater energy loss, and the secondary electron energy can therefore be effectively captured in a thin film. This enhanced stopping phenomenon can be used not only to increase the efficiency of sensors at higher energies, but it can also be exploited to make lightweight thin‐film radiation shields, particularly for charged particles, which is relevant for mass‐restrictive applications such as spacecraft and aircraft.

**Figure 11:**
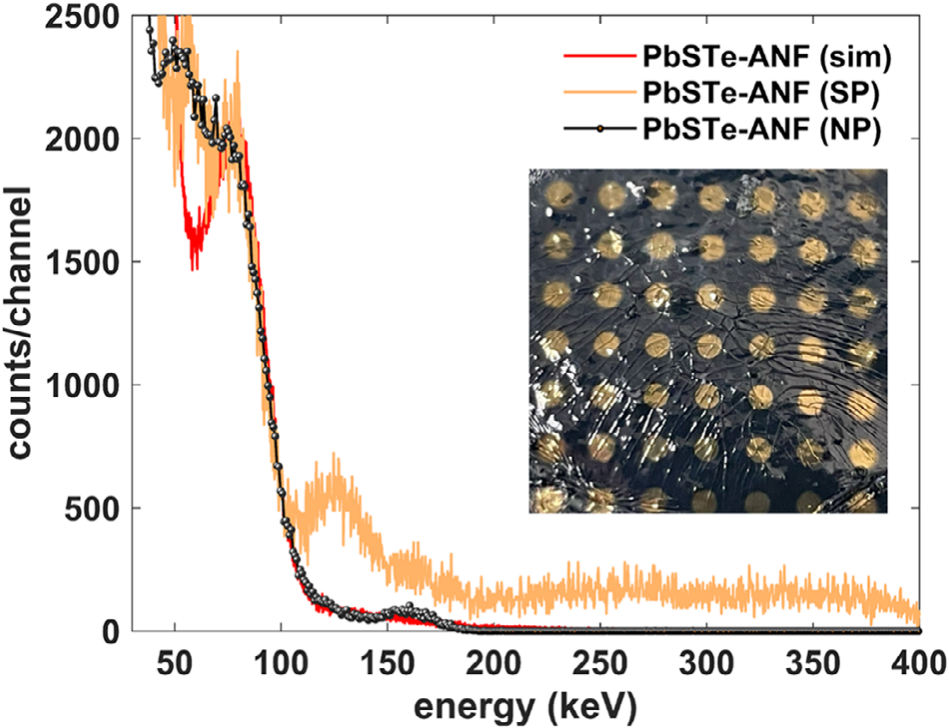
The ^133^Ba spectrum (in black) of the PbS_0.5_Te_0.5_ 60 °C nanocrystallite or nanoparticle (*PbSTe (NP)*) compared to a simulated response (red) as well as that for a PbS_x_Te_y_ supraparticle detector (*PbSTe (SP)*) of Figure 10C with similar leakage current (3.7 nA). The PbS_x_Te_y_/ANF nanocrystalline composite, with a density of 1.498 g cm^−3^ and a thickness of 105 µm, is biased to 143.9 V (leakage current: 2.7 nA), and the collection time is 48 min.

Nevertheless, the band‐gap of 3.4 eV (cf. Figure 6A) is well beyond the ≈1 eV thermal excitation barrier desired for room temperature operation, the consequence of which is smaller pulses and greater stochasticity in the pulse amplitude. The challenge of implementing the larger PbS_x_Te_y_ NPs into a pulse‐mode photonic sensor is suggested in the I–V characteristics of the loaded composites in Figure 9. Namely, the leakage current is typically 10's of nanoamperes for typical bias voltages in the 100–300 V range, compared to dark currents less than 0.1 nA for the PbS_x_Te_y_ supercluster solids used to measure the spectra in Figure 10. The higher leakage current can be attributed, in part, to the lower band‐gap of the nanocrystallites that accompanies weaker confinement. Thus, the morphological change of the nanoparticles can affect both the signal and noise, the relative competition of which determines the spectral sensitivity. As shown in **Figure** **11**, the nanocrystallite detector response (*PbSTe NP*) is similar than that generated by the PbS_x_Te_y_ supraparticles (*PbSTe SP*) in Figure 10, if detectors with similar leakage current are compared. This comparison reveals that the leakage current is high enough that it governs the resolution of the material rather than any changes associated with the band‐gap. We have previously realized excellent energy resolution with PbSe NPs with <1 eV size‐dependent band‐gap,^[^ ^30^ ^]^ and therefore expect that similar performance can be realized if the NP surface is better passivated, which we typically accomplish via either cation doping or metallic NP inclusion, both of which can fill surface‐induced inter‐band states.^[^ ^3^ ^]^

## 3 Conclusion

The development of a synthesis method to create scalable charge‐percolation nanoscale networks can inform the material design for photonic sensors across the wavelength range, as well as any device structure for which a de‐coupling of the phononic and electronic modes enhances its performance. For the PbS_x_Te_y_ system in particular, when the NPs are bonded within an aramid nanofiber network such that charge‐percolation is realized, the resulting solid can be implemented into an X‐ray/γ‐ray sensor with good energy resolution. Although comparable in resolution to that of a commercial single‐crystalline CdTe sensor, the 2.8 keV energy uncertainty, at 81 keV, is five times worse than that observed for the CdTe/Ag‐ANF nanocomposite.^[^ ^3^ ^]^ For the lead‐based *nanocluster* composites, this is due to smaller signals, as governed by the relative band‐gaps (3.4 eV for PbS_x_Te_y_ vs. 2.2 eV for CdTe^[^ ^3^ ^]^). For the PbS_x_Te_y_ nanocrystallites with poorer surface passivation and a smaller (1 eV) band‐gap, the leakage current governs the resolution performance. Based on our work with PbSe,^[^ ^31^ ^]^ our expectation is that inadequate passivation of the ligand‐mediated surface states, rather than the magnitude of the band‐gap, is the reason that the resolution doesn't improve.

From an efficiency standpoint, both cadmium and lead‐based nanosemiconductor composites exhibit unexpectedly high stopping of the secondary electrons. Specifically, the presence of high‐energy γ‐ray spectral features shows that the nanostructured solid is a more effective collector of charged‐particle energy information than a homogeneous‐equivalent solid. This latter observation suggests a new material modality for charged‐particle shielding in which enumerable nanoscale scattering sites can be distributed throughout the solid to yield thinner, lighter‐weight radiation shielding. For future applications such as thermoelectrics, the sluggishness of the lattice vibrational modes coupled with fast electron transport makes lead telluride and its alloys the materials of choice for mid‐temperature thermoelectrics. The thermoelectric figure‐of‐merit can be further enhanced via nanostructuring the material because enhanced phonon scattering can further retard thermal conductivity. Thus, the presence of interfaces and gaps is not a hindrance to device performance in these contexts, but a means to realize new capabilities relative to charge‐transport and information extraction.

## 4 Experimental Section

### Reagents

Polyvinylpyrrolidone (PVP), average mol wt 10000, Sigma‐Aldrich; lead(II) nitrate (Pb(NO_3_)_2_), 99.999% trace metal basis, Sigma‐Aldrich; thiolglycolic acid (C_2_H_4_O_2_S – TGA), ≥99%, Sigma‐Aldrich; sodium hydroxide (NaOH), pellets, ≥97.0%, ACS reagent; hydrazine hydrate solution (N_2_H_4_. H_2_O), puriss. p.a., 24%–26% in H_2_O (RT), Sigma‐Aldrich; sodium tellurite (Na_2_TeO_3_), – 100 mesh, 99%, Sigma‐Aldrich; potassium hydroxide (KOH), pellets, ≥85%, Sigma‐Aldrich; dimethyl sulfoxide (C_2_H_6_OS – DMSO), ACS reagent, ≥99.9, Sigma‐Aldrich; kevlar threads, DuPont; Deionized water.

### Lead Sulfide Telluride Syntheses by Nonclassical Nucleation

PbS_x_Te_y_ nanoparticles were prepared in an aqueous solution under ambient conditions. First, 200 mL of deionized water was added to a 250 mL three‐neck round‐bottom flask. A 3.5 cm oval stir bar was used in this experiment to facilitate the reaction. First, 0.640 g (1.932 mmol) of Pb(NO_3_)_2_ was added to the flask and stirred for 15 min to fully dissolve the lead precursor. 300 µL of TGA was added dropwise to the solution and magnetically stirred. Nitrogen gas was bubbled through the TGA‐Pb complex solution while it was magnetically stirred for 45 min to degas the aqueous solution. 0.438 g of Na_2_TeO_3_ (1.976 mmol) was then added to the stirring solution. The pH of the solution was then immediately adjusted to 11.2 by dropwise addition of 1m NaOH. The hydroxyl group acted as a weak reducing agent, and the color of the solution gradually turned from clear to dark brown as the tellurium was reduced and nanoparticles nucleate. This solution was allowed to stir at room temperature for an hour to allow the solution to reach equilibrium. The flask was then inserted into a silicone oil bath under reflux conditions to initiate the nonclassical nucleation into cubic particles.

### Lead Sulfide Telluride Nanoparticles Synthesis with PVP

**Figure 12:**
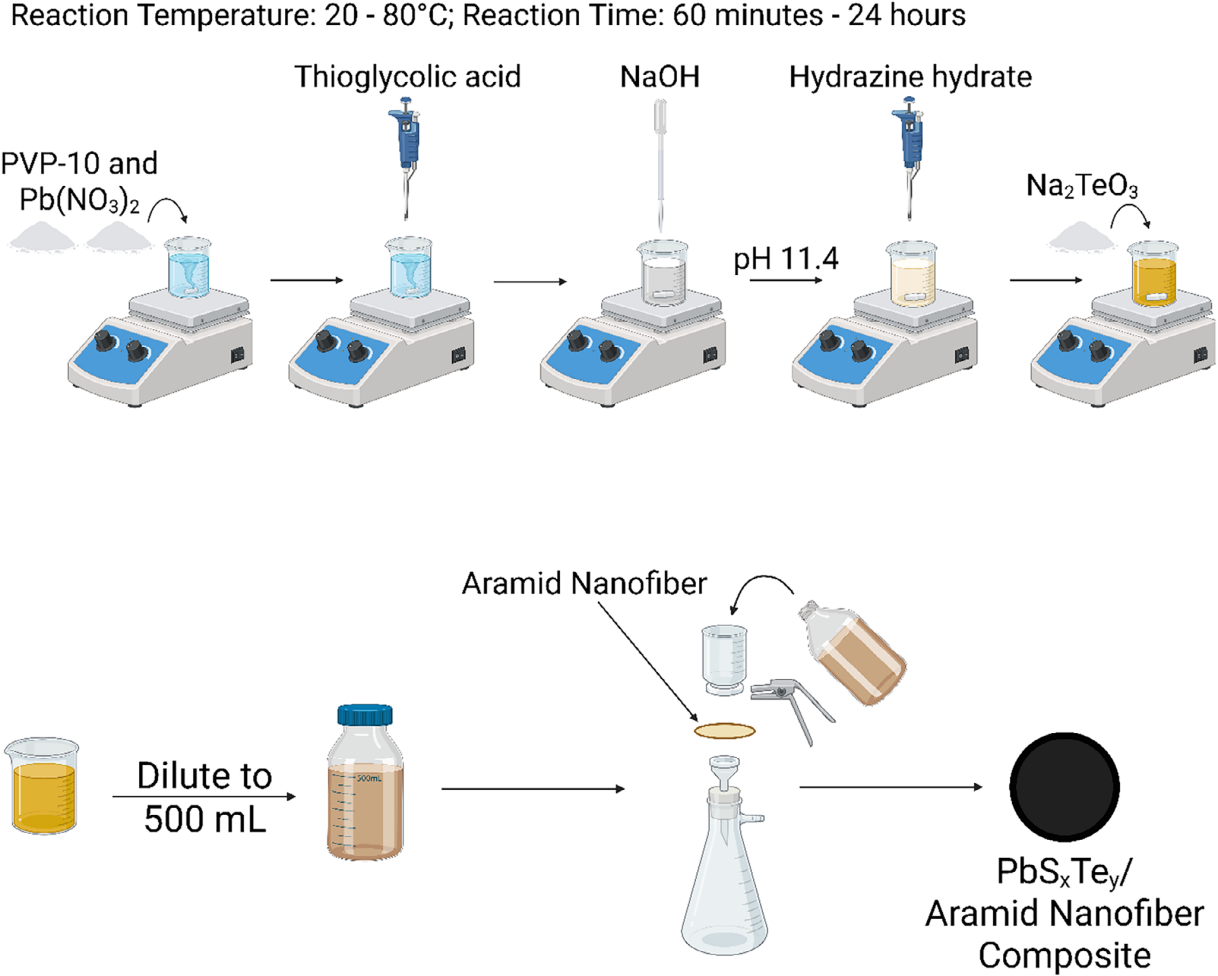
Experimental scheme of the PbS_x_Te_y_ NPs synthesis and PbS_x_Te_y_/ANF composite fabrication created with BioRender.^[^ ^68^ ^]^

The synthesis steps are illustrated in the schematic diagram in **Figure** **12**. PbS_x_Te_y_ nanoparticles were prepared in an aqueous solution under ambient conditions. Similarly, first, 200 mL of deionized water was added to a 250 mL three‐neck round‐bottom flask. The reaction temperature of the flask can be controlled from 20 to 80 °C using a heating mantle, and the stir speed was set to 700 rpm using a magnetic stirrer. In comparison with the previous method, this synthesis pathway differs in the inclusion or exclusion of PVP as well as the precursor concentrations. For this PVP‐inclusion route, in the flask, 5 g (0.5 mmol) of PVP was added and stirred for 5 min. The PVP helped to stabilize and chemically isolate the NPs and allowed them to grow to larger diameters than those superclusters that were produced via the PVP‐absent route. Subsequently, 0.1656 g (0.5 mmol) of Pb(NO_3_)_2_ was added to the flask and stirred for 10 min. Simultaneously, TGA was removed from the refrigerator and allowed to warm to room temperature. Then, 70 µL of TGA was added dropwise to the solution. The solution was left to react for another 5 min. In the last minute, the solution will get cloudier to a fully white color, indicating the full interaction between Pb and TGA.

A NaOH solution (1m NaOH) was then added dropwise to the solution until a pH of 11.4 was reached. With each dropwise addition of the base, the color of the solution becomes increasingly clear until it turns translucent. This phenomenon could be a result of the full deprotonation of all carboxylic acid and thiol sites of TGA. The solution's pH was also more sensitive at this range until it reached 11, where it slowly increased up to 11.4 with each base addition, possibly manifesting a solution buffer stage. Immediately after the solution reached a pH of 11.4, different N_2_H_4_ concentrations ranging from 0.64 mL (5 mmol – 10x), 2.5 mL (20 mmol – 40x) to 4.5 mL (35 mmol – 70x) or 6.4 mL (50 mmol – 100x) were added at once in the solution. Last, immediately after the addition of N_2_H_4_, 0.2216 g of Na_2_TeO_3_ (1 mmol) is added to the flask. The flask was then sealed with rubber stoppers and left to react for the desired reaction time. A reflux condenser column was also attached to the flask if the reaction temperature was 80 °C. The color of the solution will typically remain a dark brown/black color after the reaction is completed.

### Aramid Nanofiber Synthesis

Aramid nanofiber (ANF) is prepared by modifying the procedure previously reported by Yang et al. 2011.^[^ ^69^ ^]^ To make 2 wt.% ANF, 4 g of Kevlar thread and 4 g of ground KOH pellets are dissolved in 180 mL DMSO. The solution is stirred for 2 weeks to ensure that the Kevlar undergoes complete dissolution. The resulting thick, red, viscous dispersion is then made into a thin film or a thicker solid piece by two separate hydrolysis pathways.

If a spin‐cast thin film was the desired matrix for the composite, the as‐prepared thick, red, viscous dispersion above was then spun‐cast (10 s spread at 100 rpm and 15 s spin at 200 or 325 rpm) upon a 5 cm‐diameter circular glass slide. The glass slide was then immediately submerged in deionized water to remove the DMSO and protonate the ANF. The thin film was typically left in water for ≈10 min until its color turned opaque white, indicating complete hydrolysis.

In case a larger, thicker (5 mm thickness) piece of solid was the desired matrix, the viscous ANF was then instead poured into a 3D‐printed, round mold with multiple tiny pores on the side (2 mm diameter) and a diameter of 6 cm (Figure S22, Supporting Information). The mold was then put into a water bath with a plastic petri dish cover on top of it to avoid ANF leaking out of the mold. A weight was then put on the plastic petri dish cover when water was filled in the bath up to the point when the whole mold was submerged. Likewise, the viscous ANF was allowed to hydrolyze until its color turned to white, which typically will take approximately 2 weeks.

### Synthesis of Ag NPs

Citrate‐stabilized silver nanoparticles were synthesized according to a procedure previously described.^[^ ^70^ ^]^ 200 mL of ePure water was added to a 250 mL 3‐neck flask. 0.294 g of trisodium citrate dihydrate and 0.034 g of tannic acid were added to the flask. The flask was brought boiling by heating in a silicone oil bath, and refluxed under magnetic stirring. After the solution had reached 100 °C for 15 min, 8 mL of a 25 mm solution of silver nitrate was injected into the flask. The flask continued to reflux under stirring for another 15 min, until the solution turned a yellow‐brown color. The solution was allowed to cool, and the nanoparticles were purified by centrifugation at 15k × g for 30 min, to separate excess tannic acid. The collected Ag NPs were redispersed in ePure water.

### Preparation of Nanocomposite Film

After colloidal synthesis, all the solutions were quenched by dilution using cold deionized water to 500 mL. Using a vacuum filtration set up, the diluted solution was fluxed through an aramid nanofiber hydrogel, which acted as a scaffold for the nanoparticles. In case the solution was flown through a thin film, a filter paper was put below the ANF in the vacuum filtration setup to enhance the integrated capacity of the composite. This insertion of a filter paper was, on the other hand, unnecessary if a thick ANF was used.

### Materials Characterization

Characterization of the PbS_x_Te_y_ nanoparticles were performed using scanning and transmission electron microscopes (SEM and TEM), energy dispersive spectroscopy (EDS), and X‐ray diffraction (XRD). SEM images with EDS spectra were collected with a JSM‐IT500HR InTouchScope scanning electron microscope, which had a resolution of 1.5 nm (0.5–30 kV). TEM images collected with a 300 kV FEI Tecnai TEM, which had a lattice resolution of 0.1 nm for CTEM and a Thermo Fisher Talos F200X G2 S/TEM, which had CTEM point resolution at 0.25 nm and STEM <0.16 nm (80 and 200 kV). XRD spectra were taken using the Rigaku SmartLab X‐ray Diffractometer (Cu Kα, 40 kV, 50 mA, step 0.01°, 5.0°/min, incident slit 1/2°, receiving slit 20.000 mm). Optical measurements of the colloids were collected using a JASCO V‐770 UV–vis‐NIR Spectrophotometer (Czerny‐Turner mount, single monochromator, double beam). The loading of the PbS_x_Te_y_/ANF composite was investigated using a Discovery 5500 Thermogravimetric Analysis instrument (Ramp 10°C min^−1^ from RT to 700 °C) with argon flow. Specifically, each composite after fabrication was cut into small pieces whose weights added up to 5–10 mg. These pieces were then inserted into the analyzer that performed a heat ramp from room temperature (25 ± 5 °C) to 700 ± 5 °C.

### Current/Voltage (I/V) Measurements

**Figure 13:**
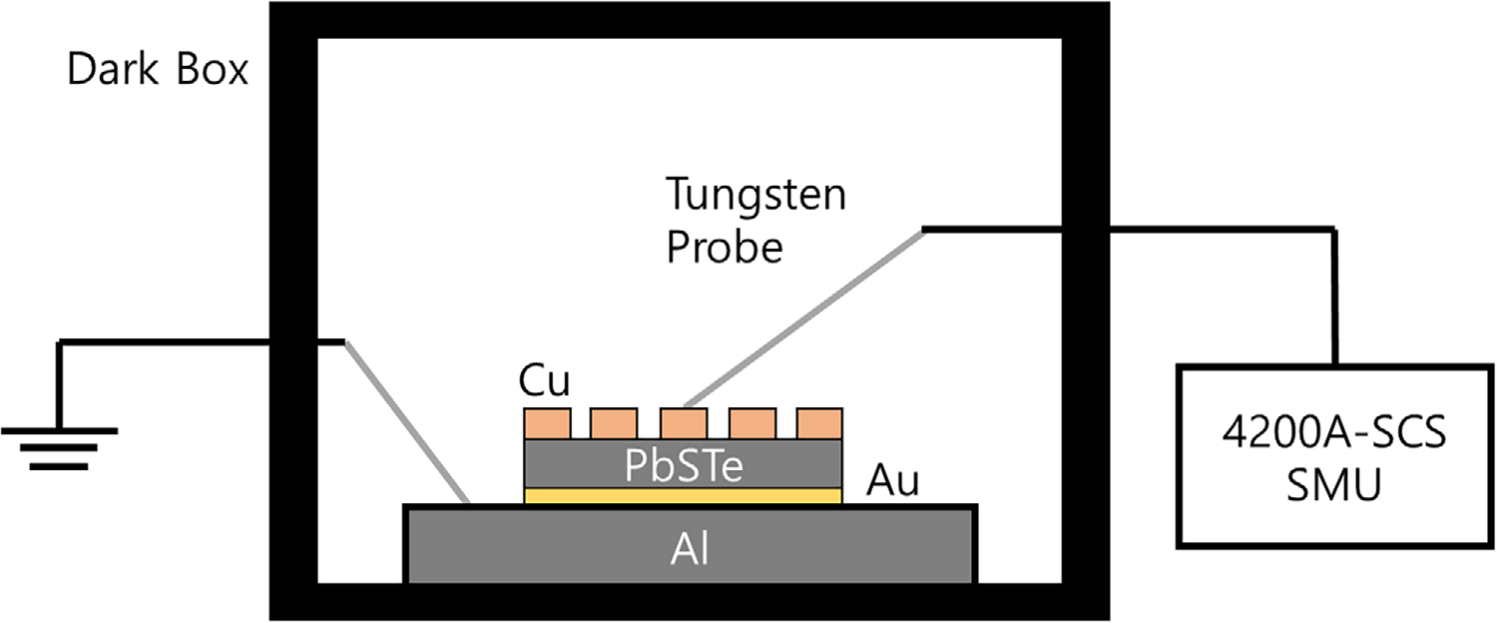
Experimental setup for I/V measurement.

The 4200A‐SCS semiconductor parameter analyzer from Keithley was used for I/V measurements. The samples were deposited with evaporated 300 nm thick electrodes consisting of copper and gold on the top and bottom side, respectively. The deposition process was done with an Angstrom Engineering Evovac Evaporator in the Lurie Nanofabrication Facility (LNF) at the University of Michigan. **Figure** **13** shows the experimental setup for the I/V measurement. The measurements were done in the dark box to prevent photoexcitation of the samples. Voltage was applied from ‐10 V to +10 V with 0.1 V linear steps. In addition, sweep delay and hold time were 0.5 and 1 s for each to avoid transient charging in the samples.

### γ‐ray Interaction Simulations

For Figure 10C, the dimensions of the polygonal solid (2.3 × 3.5 × 2.2 mm^3^) were measured with calipers, and the density (0.606 g cm^−3^) was measured by dividing the mass (0.0109 g) by the volume. For the simulated response of Figure 11, the density (1.498 g cm^−3^) and composition were similarly measured. The relative atomic fraction of the inorganic (4.1% Pb, 6.0% Te) and organic (39.5% C, 38.3% O, 12.1% Na) constituents was derived from energy dispersive X‐ray spectroscopy (EDS). The MCNP simulation included the target, an assumed point ^133^Ba source 1 cm distant from the source, and the aluminum block upon which the sensor was placed to simulate the effects of aluminum backscatter. The ^133^Ba energy distribution consisted of the following peaks (in MeV): 0.00467, 0.03063, 0.03097, 0.03492, 0.03499, 0.03525, 0.035822, 0.035907, 0.035972, 0.053, 0.0796, 0.0809,0.1606, 0.2232, 0.2763, 0.3028, 0.3560, 0.3838, with the following relative intensities: 0.03725, 0.07934, 0.1464, 0.01427, 0.01427, 0.01427, 0.00347, 0.00347, 0.00347, 0.00502, 0.0263, 0.3331, 0.00638, 0.0045, 0.0713, 0.1831, 0.6205, 0.0894. In general, a total number of 3×10^9^ photons were simulated per run and the F8 (energy deposition) tally was used to capture the spectrum. The subsequent charge electron‐hole transport within the sensor wasn't included as did in Joglekar et al. (2019)^[^ ^66^ ^]^ because of negligible charge trapping within the sensor, as evidenced via the sharp rise (< 1 µs) time and symmetric energy peaks.

### γ‐ray Interaction Measurements

A 31.55 MBq ^133^Ba X‐ray and γ‐ray disk source (radius 5 mm) was placed upon a polypropylene stand and separated from the sensor by 2 cm. Both the source and detector were placed within a vacuum chamber test box that was evacuated during measurements. The sensor sat upon an aluminum test stand and was contacted on its top surface via an aluminum probe tip. The γ‐ray spectra were collected with 550 eV charge‐sensitive preamp, an Ortec 572A shaping amplifier (shaping time: 500 ns), and an Ortec Maestro multi‐channel analyzer (MCA). The raw pulse height data collected by the MCA are channel numbers between 1 and 8192 that are derived from shaped pulses with voltage amplitudes between 0 and 10 V. The energetic positions of are identified with assistance from the MCNP simulations. The same readout chain and environmental conditions (e.g. room‐temperature) were used for the commercial CdTe detector (from Acrorad). In general, the ≈10 times greater thickness of the CdTe detector results in commensurately lower detector capacitance and therefore lower intrinsic preamp noise. Thus, the spectral comparisons represent a conservative comparison of the degree to which the *intrinsic* performance is improved compared to that of the commercial detector. This is even more so the case for the HPGe results for which the preamp (Canberra 2002C) was cooled via the cold‐finger into the LN_2_ reservoir. On the other hand, the spectral comparisons are intended to exhibit the *intrinsic* resolution achievable with the novel material by comparing it with a state‐of‐the art commercial detection medium. As the volumes of the commercial detectors (particularly the 3″ HPGe solid) are much larger, the spectral comparisons do not imply similar engineering maturity in terms of achieving minimal charge trapping over inches of transport length.

### Statistical Analysis

To derive the approximate average diameter of the nanoparticles as reported in Figure 4, 50–100 of clearly visible nanoparticles are randomly sampled at 10–20 randomly selected fields on the microscope window. The diameter data are represented as the mean ± standard deviation of all independent particles measured on the SEM views.

## Conflict of Interest

The authors declare no conflict of interest.

## Author Contributions

M.H. conceived the research based on joint work with G.K. D.V. developed the original recipes and performed the synthesis, SEM imaging, STEM imaging, and absorbance of the pure TGA recipe. A.D.U. modified the recipes and along with A.M.U., M.H. and D.V., fabricated the sensing composites. A.D.U. also innovated the PVP inclusion to modify the NP size. From the PVP recipe, V.‐D.L. performed the systematic PbS_x_Te_y_ syntheses, the SEM imaging with its EDS elemental mapping and quantification analysis, the XRD analysis, and the thermogravimetric analysis. I.‐H. H. performed the absorbance spectroscopy, the TEM imaging, and along with V.‐D.L., performed the IV characterization. M.H. performed the radiation spectroscopy and radiation‐interaction modeling. V.‐D.L., D.V., and M.H. prepared the manuscript.

## Supporting information



Supporting Information

## Data Availability

The data that support the findings of this study are available in the supplementary material of this article.

## References

[1] C. F. Landes , M. Braun , M. A. El‐Sayed , J. Phys. Chem. B. 2001, 105, 10554.

[2] D.‐R. Jung , J. Kim , C. Nahm , H. Choi , S. Nam , B. Park , Electron. Mater. Lett. 2011, 7, 185.

[3] D. A. Vecchio , M. D. Hammig , N. A. Kotov , ACS Nano. 2025, 19, 11924. 40106385 10.1021/acsnano.4c15939

[4] S. Kasap , J. B. Frey , G. Belev , O. Tousignant , H. Mani , J. Greenspan , L. Laperriere , O. Bubon , A. Reznik , G. DeCrescenzo , K. S. Karim , J. A. Rowlands , Sensors. 2011, 11, 5112. 22163893 10.3390/s110505112 PMC3231396

[5] W. Zhao , D. C. Hunt , K. Tanioka , J. A. Rowlands , Nucl. Instrum. Methods Phys. Res. A: Accel. Spectrom. Detect. Assoc. Equip. 2005, 549, 205.

[6] J. Stavro , A. H. Goldan , W. Zhao , J. Med. Imaging. 2018, 5, 1. 10.1117/1.JMI.5.4.043502 PMC6206442 30840737

[7] H. Huang , S. Abbaszadeh , IEEE Sensors J. 2020, 20, 1694.

[8] O. Semeniuk , O. Grynko , G. Juska , A. Reznik , Sci. Rep. 2017, 7, 13272. 29038544 10.1038/s41598-017-13697-2 PMC5643314

[9] O. Semeniuk , O. Grynko , G. Decrescenzo , G. Juska , K. Wang , A. Reznik , Sci. Rep. 2017, 7, 8659. 28819287 10.1038/s41598-017-09168-3 PMC5561065

[10] E. Pineau , O. Grynko , T. Thibault , A. Alexandrov , A. Csík , S. Kökényesi , A. Reznik , Sensors. 2022, 22, 5998. 36015758 10.3390/s22165998 PMC9412672

[11] A. Saha , L. J. Guo , M. D. Hammig , Nucl. Instrum. Methods Phys. Res. A: Accel. Spectrom. Detect. Assoc. Equip. 2024, 1066, 169588.

[12] A. Nathan , B. Park , Q. Ma , A. Sazonov , J. A. Rowlands , Microelectron. Reliab. 2002, 42, 735.

[13] D. E. Carlson , C. R. Wronski , In (Eds: T. Markvart , L. Castañer ), Practical Handbook of Photovoltaics, Elsevier, Amsterdam 2003, 281.

[14] M. Menichelli , L. Servoli , N. Wyrsch , Front. Phys. 2022, 10, 943306.

[15] M. J. Large , M. Bizzarri , L. Calcagnile , M. Caprai , A. P. Caricato , R. Catalano , G. A. P. Cirrone , T. Croci , G. Cuttone , S. Dunand , Phys. Med. Biol. 2023, 68, 135010. 10.1088/1361-6560/acdb43 37267990

[16] H. Wei , J. Huang , Nat. Commun. 2019, 10, 1066. 30842411 10.1038/s41467-019-08981-w PMC6403296

[17] F. Liu , R. Wu , J. Wei , W. Nie , A. D. Mohite , S. Brovelli , L. Manna , H. Li , ACS Energy Lett. 2022, 7, 1066.

[18] K. Sakhatskyi , A. Bhardwaj , G. J. Matt , S. Yakunin , M. V. Kovalenko , Adv. Mater. 2025, 37, 2418465. 10.1002/adma.202418465 40317506

[19] J. Liu , P. Liu , T. Shi , M. Ke , K. Xiong , Y. Liu , L. Chen , L. Zhang , X. Liang , H. Li , S. Lu , X. Lan , G. Niu , J. Zhang , P. Fei , L. Gao , J. Tang , Nat. Commun. 2023, 14, 5352. 37660051 10.1038/s41467-023-40620-3 PMC10475073

[20] M. Ruggieri , E. Colantoni , E. Marconi , A. Fabbri , P. Branchini , L. Colace , L. Tortora , A. De lacovo , ACS Appl. Electron. Mater. 2023, 5, 5642.

[21] G. N. Ankah , P. Büchele , K. Poulsen , T. Rauch , S. F. Tedde , C. Gimmler , O. Schmidt , T. Kraus , Org. Electron. 2016, 33, 201.

[22] A. K. Tam , O. Boyraz , J. Unangst , P. Nazareta , M. Schreuder , M. Nilsson , Radiat. Meas. 2018, 111, 27.

[23] J. Boo , I. H. Han , G. Kim , J. Radiat. Prot. Res. 2025, 50, 63.

[24] J. E. Murphy , M. C. Beard , A. G. Norman , S. P. Ahrenkiel , J. C. Johnson , P. Yu , O. I. Mićić , R. J. Ellingson , A. J. Nozik , J. Am. Chem. Soc. 2006, 128, 3241. 16522105 10.1021/ja0574973

[25] T. J. Zhu , Y. Q. Liu , X. B. Zhao , Mater. Res. Bull. 2008, 43, 2850.

[26] R. Miranti , D. Shin , R. D. Septianto , M. Ibáñez , M. V. Kovalenko , N. Matsushita , Y. Iwasa , S. Z. Bisri , ACS Nano. 2020, 14, 3242. 32073817 10.1021/acsnano.9b08687

[27] X. Zhu , Z. Liu , G. Shi , J. Gu , W. Wang , W. Ma , J. Mater. Res. Technol. 2017, 33, 418.

[28] H.‐Q. Liu , F.‐X. Hao , J. Guo , Y.‐J. Gu , Q.‐K. He , H.‐Z. Cui , in 2012 Int. Conf. on Manipulation, Manufacturing and Measurement on the Nanoscale (3M‐NANO) , IEEE, Xi'an, China 2012, 382.

[29] Y. Y. Wang , K. F. Cai , X. Yao , J. Solid State Chem. 2009, 182, 3383.

[30] M. D. Hammig , in (Eds M. Nenoi ) Current Topics in Ionizing Radiation Research, IntechOpen, London, 2012, 557.

[31] B. J. Davis , M. D. Hammig , ACS Appl. Nano Mater. 2021, 4, 6936.

[32] D. A. Vecchio , M. D. Hammig , X. Xiao , A. Saha , P. Bogdan , N. A. Kotov , Adv. Mater. 2022, 34, 2201313. 10.1002/adma.202201313 35403264

[33] J. A. Kulesza , T. R. Adams , J. C. Armstrong , S. R. Bolding , F. B. Brown , J. S. Bull , T. P. Burke , A. R. Clark , R. A. Forster III , J. F. Giron , A. S. Grieve , C. J. Josey , R. L. Martz , G. W. McKinney , E. J. Pearson , M. E. Rising , C. J. Solomon Jr , S. Swaminarayan , T. J. Trahan , C. A. Weaver , S. C. Wilson , A. J. Zukaitis , MCNP® Code Version 6.3.1 Theory & User Manual. Los Alamos National Laboratory Tech. Rep. LA‐UR‐24‐24602 Rev. 1. Los Alamos, NM, USA. May 2024.

[34] K. Hummer , A. Grüneis , G. Kresse , Phys. Rev. B. 2007, 75, 195211.

[35] S. Sze , K. Ng , Physics of Semiconductor Devices, Wiley, New Jersey 2006.

[36] M. J. Berger , J. H. Hubbell , S. M. Seltzer , J. Chang , J. S. Coursey , R. Sukumar , D. S. Zucker , K. Olsen , XCOM: Photon Cross Sections Database National Institue of Standards and Technology 1998.

[37] D. F. Jackson , D. J. Hawkes , Phys. Rep. 1981, 70, 169.

[38] A. Jain , S. P. Ong , G. Hautier , W. Chen , W. D. Richards , S. Dacek , S. Cholia , D. Gunter , D. Skinner , G. Ceder , K. A. Persson , APL Mater. 2013, 1, 011002.

[39] M. Ruggieri , F. Mitri , A. Fabbri , P. Branchini , V. Graziani , L. Colace , L. Tortora , A. De lacovo , ACS Appl. Electron. Mater. 2025, 7, 5549.

[40] A. Brovko , O. Amzallag , A. Adelberg , A. Ruzin , Nucl. Instrum. Methods Phys. Res. A: Accel. Spectrom. Detect. Assoc. Equip. 2021, 1014, 165696.

[41] H. Eggers , F. Schackmar , T. Abzieher , Q. Sun , U. Lemmer , Y. Vaynzof , B. S. Richards , G. Hernandez‐Sosa , U. W. Paetzold , Adv. Energy Mater. 2020, 10, 1903184.

[42] F. Schackmar , H. Eggers , M. Frericks , B. S. Richards , U. Lemmer , G. Hernandez‐Sosa , U. W. Paetzold , Adv. Mater. Technol. 2021, 6, 2000271.

[43] H. Mescher , F. Schackmar , R. Huber , H. Eggers , M. Zuber , E. Hamann , G. Gramlich , J. Dangelmaier , Q. Zhang , A. G. Rösch , T. Zwick , G. Hernandez‐Sosa , U. W. Paetzold , U. Lemmer , npj Flex Electron. 2023, 7, 9.

[44] H. Mescher , F. Schackmar , H. Eggers , T. Abzieher , M. Zuber , E. Hamann , T. Baumbach , B. S. Richards , G. Hernandez‐Sosa , U. W. Paetzold , U. Lemmer , ACS Appl. Mater. Interfaces. 2020, 12, 15774. 32182029 10.1021/acsami.9b14649

[45] C. Xiao , F. Maddalena , Q. Wu , T. Mariyappan , F. Huang , M. He , A. Bruno , N. Mathews , C. Dang , ACS Appl. Electron. Mater. 2025, 7, 4004.

[46] Y. Lu , D. He , X. Yuan , Q. Yan , X. Shu , Z. Hu , Z. Zhang , Z. Liu , Z. Jiang , R. Xu , W. Wang , Z. Ma , T. Chen , H. Xu , F. Xu , F. Hong , H. Song , Adv. Funct. Mater. 2025, 35, 2413507.

[47] K.‐W. Huang , M.‐H. Li , Y.‐T. Chen , Z.‐X. Wen , C.‐F. Lin , P. Chen , J. Mater. Chem. C. 2024, 12, 1533.

[48] K. Sakhatskyi , B. Turedi , G. J. Matt , E. Wu , A. Sakhatska , V. Bartosh , M. N. Lintangpradiptop , R. Naphade , I. Shorubalko , O. F. Mohammed , S. Yakunin , O. M. Bakr , M. V. Kovalenko , Nat. Photonics. 2023, 17, 510.

[49] J. Cheng , C. Xue , M. Yang , X. Wang , Z. Xu , N. Li , X. Zhang , X. Feng , X. Liu , Y, L. , S. F. Liu , Z. Yang , ACS Appl. Mater. Interfaces. 2024, 16, 36649. 38961051 10.1021/acsami.4c08706

[50] D. Gebauer , A. Völkel , H. Cölfen , Science. 2008, 322, 1819. 19095936 10.1126/science.1164271

[51] F. C. Meldrum , R. P. Sear , Science. 2008, 322, 1802. 19095931 10.1126/science.1167221

[52] H.‐C. Chang , J. A. Ho , Anal. Chem. 2015, 87, 10362. 26379119 10.1021/acs.analchem.5b02452

[53] A. Yahia‐Ammar , D. Sierra , F. Mérola , N. Hildebrandt , X. L. Guével , ACS Nano. 2016, 10, 2591. 26845515 10.1021/acsnano.5b07596

[54] K. Persson , The Materials Project. 2011, https://next‐gen.materialsproject.org/.

[55] H. Yang , S. W. Finefrock , J. D. Albarracin Caballero , Y. Wu , J. Am. Chem. Soc. 2014, 136, 10242. 25003347 10.1021/ja505304v

[56] N. T. K. Thanh , N. Maclean , S. Mahiddine , Chem. Rev. 2014, 114, 7610. 25003956 10.1021/cr400544s

[57] F. Liebig , A. F. Thünemann , J. Koetz , Langmuir. 2016, 32, 10928. 27696870 10.1021/acs.langmuir.6b02662

[58] E. Piletska , H. Yawer , F. Canfarotta , E. Moczko , K. Smolinska‐Kempisty , S. S. Piletsky , A. Guerreiro , M. J. Whitcombe , S. A. Piletsky , Sci. Rep. 2017, 7, 11537. 28912505 10.1038/s41598-017-12007-0 PMC5599519

[59] W. B. Pearson , A Handbook of Lattice Spacings and Structures of Metals and Alloys, Pergamon Press, South Croydon, United Kingdom. 2013.

[60] P. Makuła , M. Pacia , W. Macyk , J. Phys. Chem. Lett. 2018, 9, 6814. 30990726 10.1021/acs.jpclett.8b02892

[61] D. R. Paschotta , RP Photon. Encycloped. RP Photonics AG 2019. https://www.rp‐photonics.com/encyclopedia.html.

[62] J. Klein , L. Kampermann , B. Mockenhaupt , M. Behrens , J. Strunk , G. Bacher , Adv. Funct. Mater. 2023, 33, 2304523.

[63] J. Lyu , X. Wang , L. Liu , Y. Kim , E. K. Tanyi , H. Chi , W. Feng , L. Xu , T. Li , M. A. Noginov , C. Uher , M. D. Hammig , N. A. Kotov , Adv. Funct. Mater. 2016, 26, 8435.

[64] Y. K. Du , P. Yang , Z. G. Mou , N. P. Hua , L. Jiang , J. Appl. Polym. Sci. 2006, 99, 23.

[65] W. Li , X. Xu , W. Li , P. Liu , Y. Zhao , Q. Cen , M. Chen , J. Mater. Res. Technol. 2019, 9, 142.

[66] S. G. Joglekar , M. D. Hammig , L. J. Guo , ACS Appl. Mater. Interfaces. 2019, 11, 33399. 31465191 10.1021/acsami.9b09381

[67] A. Thompson , D. Attwood , E. Gullikson , M. Howells , K.‐J. Kim , J. Kirz , J. Kortright , I. Lindau , Y. Liu , P. Pinatteta , A. Robinson , J. Scofield , J. Underwood , G. Williams , H. Winick , X‐Ray Data Booklet, Lawrence Berkeley National Laboratory, Berkeley 2009.

[68] V.‐D. Le , Created in BioRender. 2025, http://BioRender.com/bordk8i.

[69] M. Yang , K. Cao , L. Sui , Y. Qi , J. Zhu , A. Waas , E. M. Arruda , J. Kieffer , M. D. Thouless , N. A. Kotov , ACS Nano. 2011, 5, 6945. 21800822 10.1021/nn2014003 PMC3214697

[70] N. G. Bastús , F. Merkoçi , J. Piella , V. Puntes , Chem. Mater. 2014, 26, 2836.

