# Supplementary material for: X‐ray and γ‐ray Sensing from Aqueous‐Based Lead Sulfide Telluride Nanocomposites
Source: Small. 2025 Oct 25;21(49):e04684. doi: 10.1002/smll.202504684 (PMC12696808; doi:10.1002/smll.202504684)
Supplement: Supplementary file 1 — Supporting Information [file SMLL-21-e04684-s001.docx]

*Supporting Information* *for:*

X-ray and γ-ray Sensing from Aqueous-based Lead Sulfide Telluride Nanocomposites

Vinh-Dien Le^1^, Drew A. Vecchio^1,2,3^, Ayse D. Uyulur^1,3^, Ill-hyuk Han^2,4^, Andrei M. Ursu^1^, Geehyun Kim^4^, and Mark D. Hammig^,2,3,*^

^1^Department of Chemical Engineering, University of Michigan, Ann Arbor, MI 48109

^2^Department of Nuclear Engineering and Radiological Sciences, University of Michigan, Ann Arbor, MI 48109

^3^Amphionic LLC, Plymouth, MI 48170

^4^Nuclear Engineering, Seoul National University, Seoul, South Korea, 08826

*Email: hammig@umich.edu


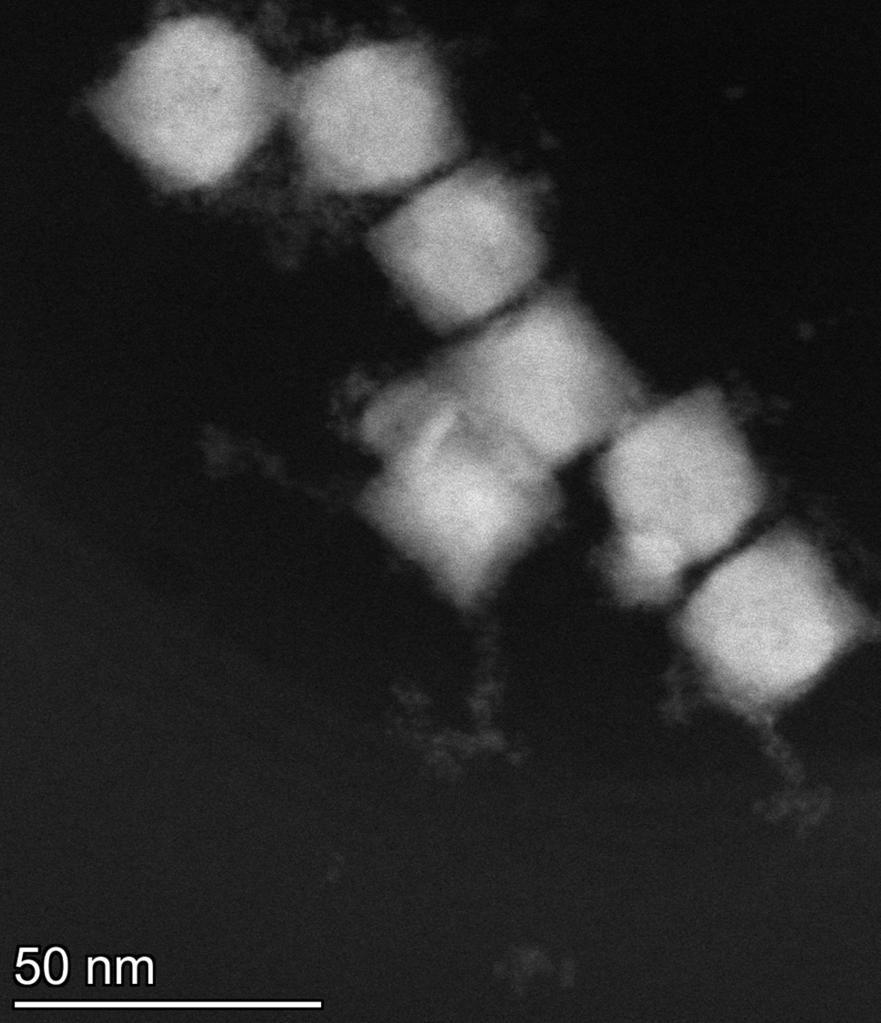

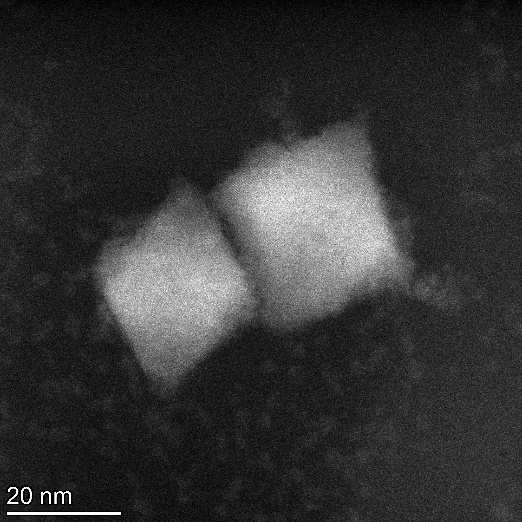

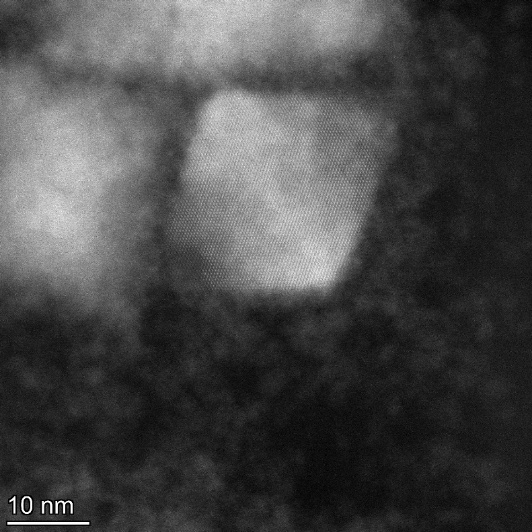


Figure S1. STEM images of PbS_x_Te_1-x_ samples synthesized with 6.4 mL N_2_H_4_ (100x) at 60°C using for 60 minutes.


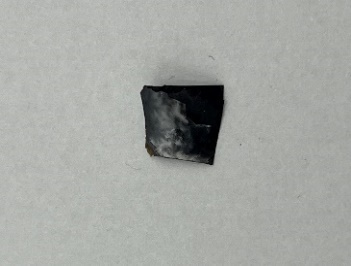

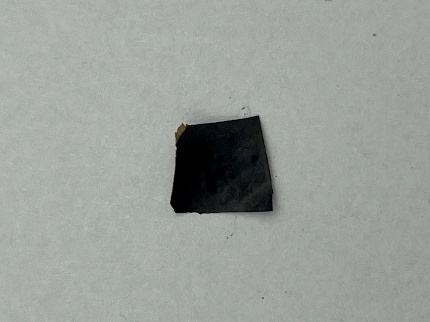

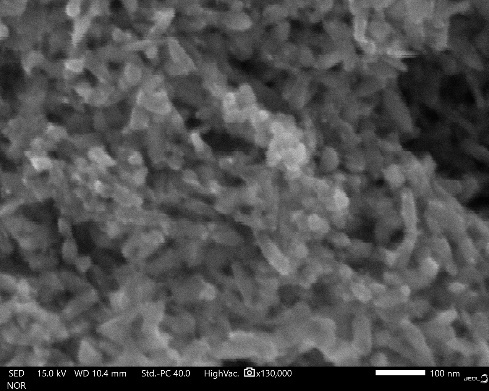

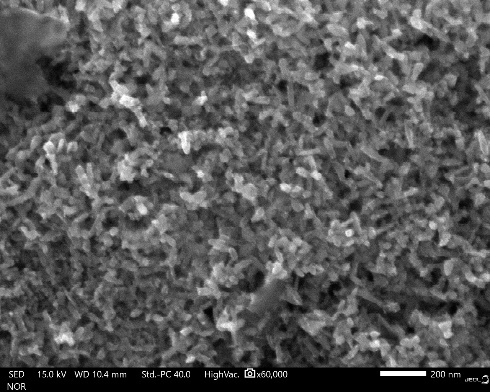

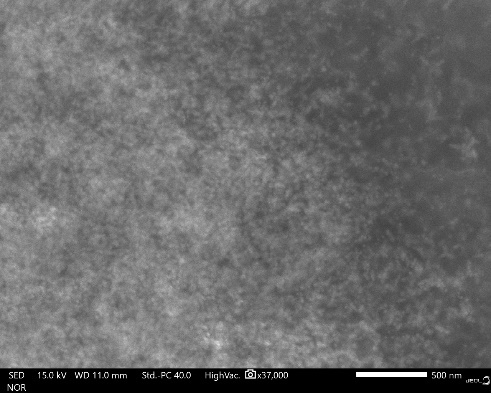

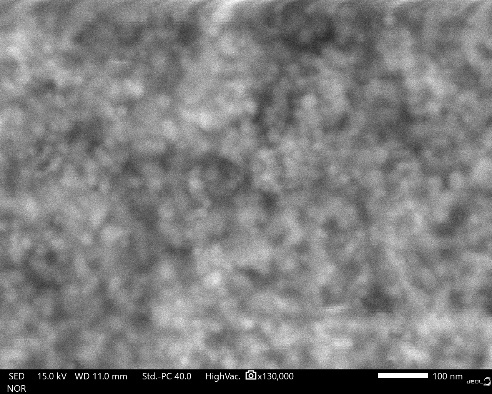


Figure S2. SEM images of PbS_x_Te_1-x_ samples synthesized with 6.4 mL N_2_H_4_ (100x) at 80°C for 60 minutes. The top images are from the top side of the composite while the bottom images are from the bottom side of the composite. The insets in some of the pictures are macroscale imaging of how the composites look.


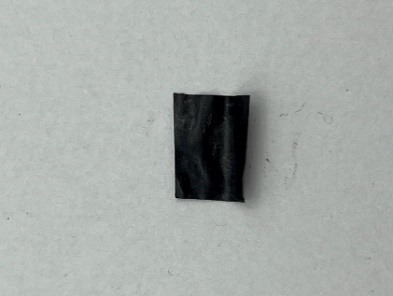

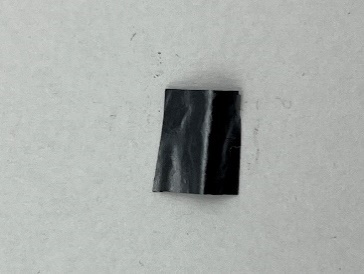

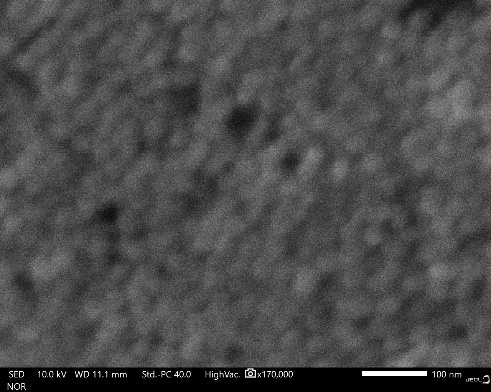

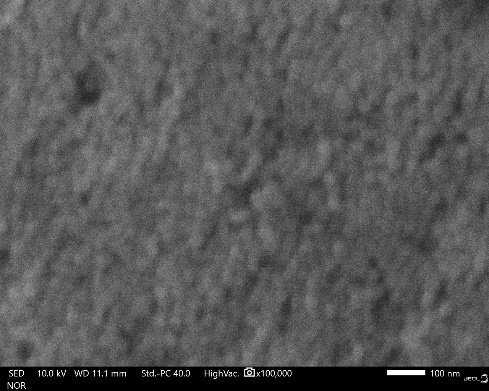


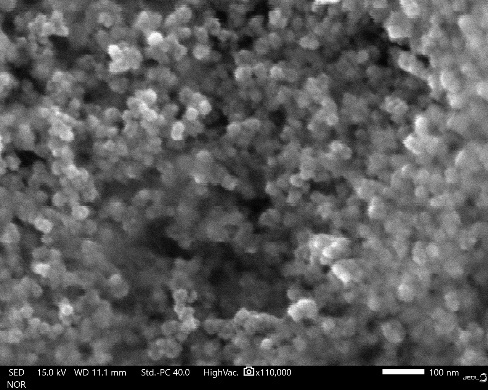

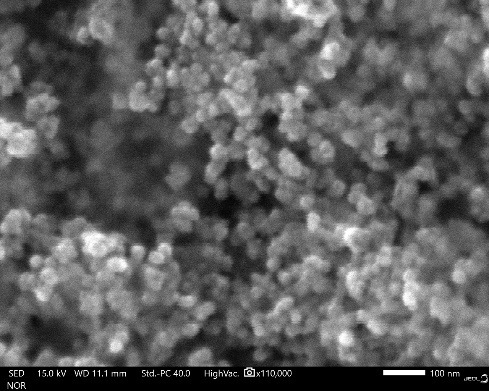


Figure S3. SEM images of PbS_x_Te_1-x_ samples synthesized with 6.4 mL N_2_H_4_ (100x) at 60°C for 90 minutes. The top images are from the top side of the composite while the bottom images are from the bottom side of the composite. The insets in some of the pictures are macroscale imaging of how the composites look.


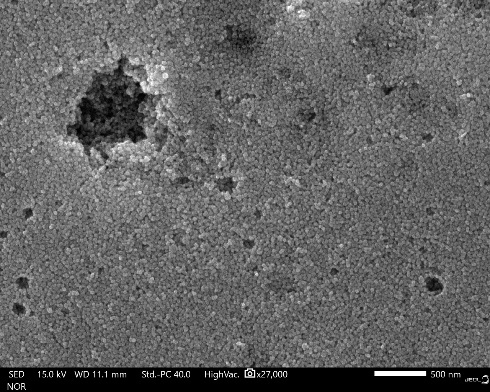

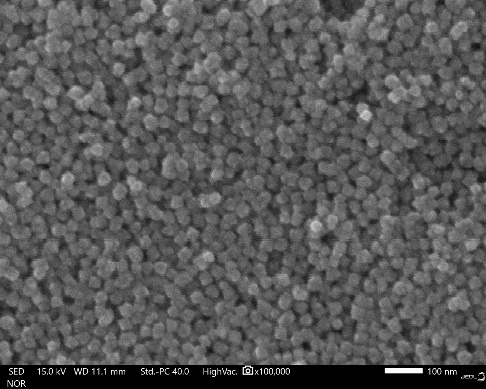

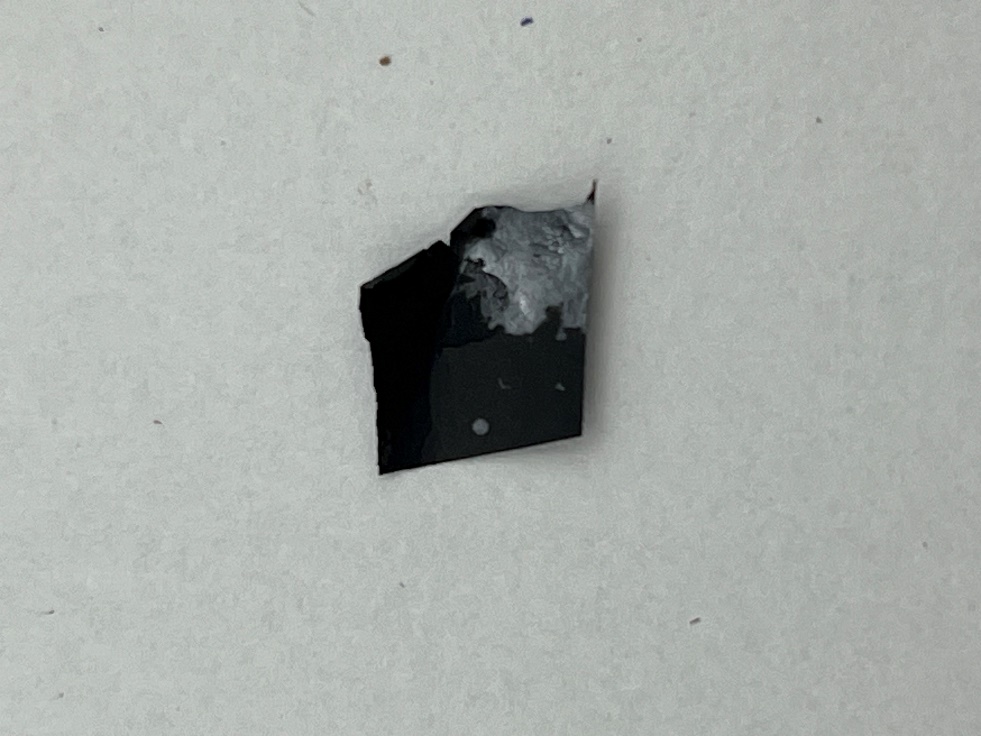

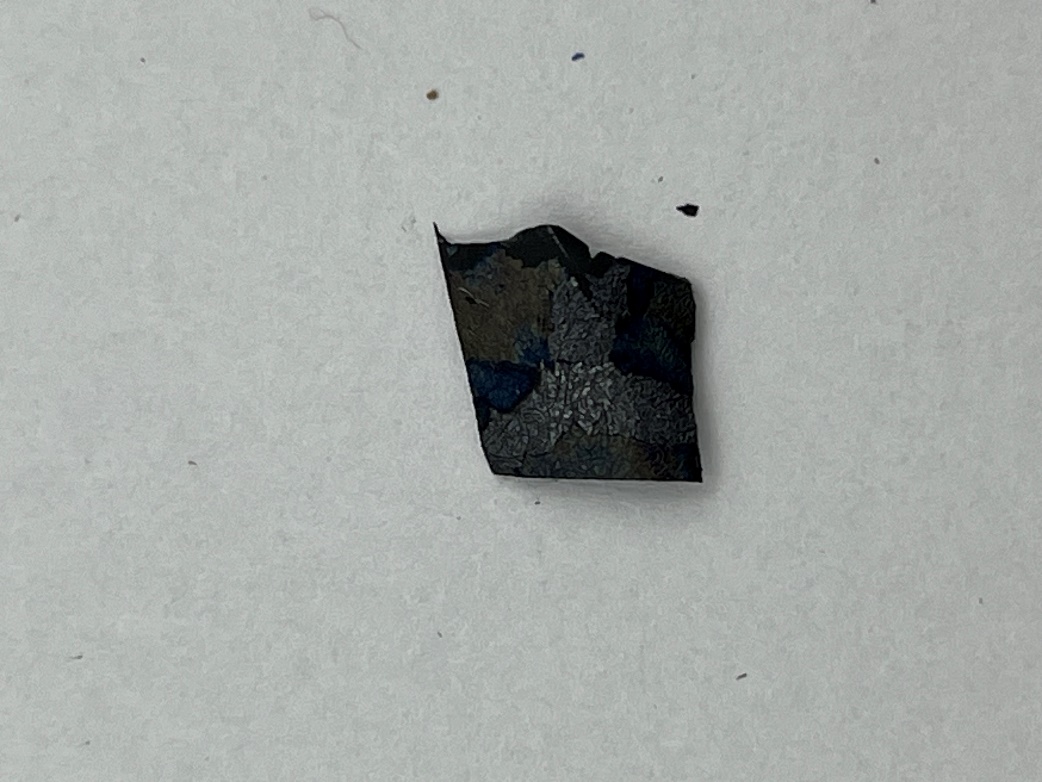


Top Bottom

Figure S4. SEM images of PbS_x_Te_1-x_ samples synthesized with 6.4 mL N_2_H_4_ (100x) at 60°C for 120 minutes. The top images are from the top side of the composite while the bottom images are from the bottom side of the composite. Note that no particles are spotted via SEM from the bottom side of this composite. The bottom images are macroscale imaging of how the composites look.


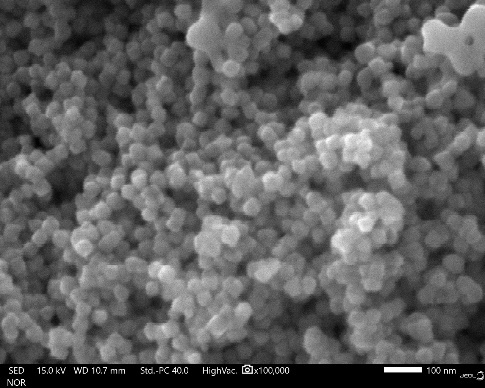

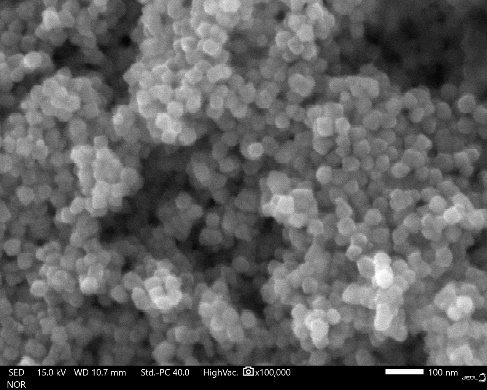

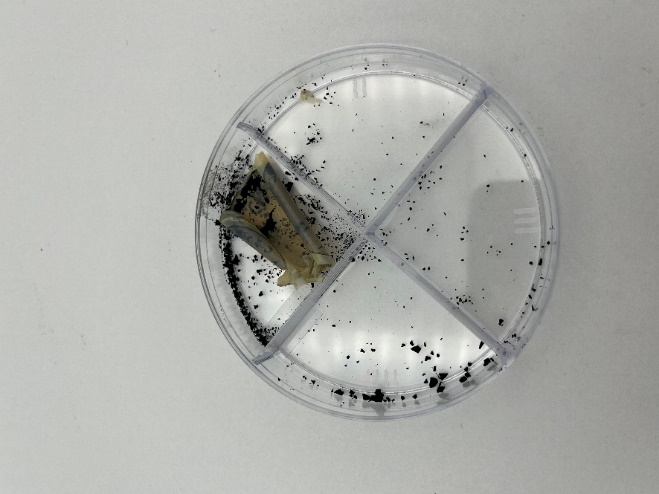


Figure S5. SEM images of PbS_x_Te_1-x_ samples synthesized with 6.4 mL N_2_H_4_ (1x) at 60°C for 24 hours. The top images are from the top side of the composite. Note that no composite is successfully made for this sample as the particles surface loaded on the ANF and then crack apart into powder as illustrated by the bottom macroscale images.


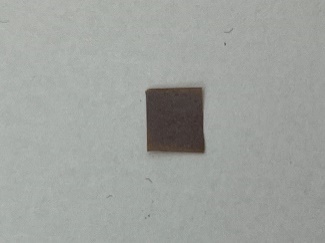


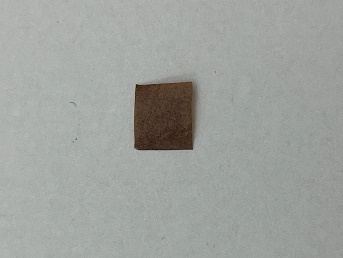

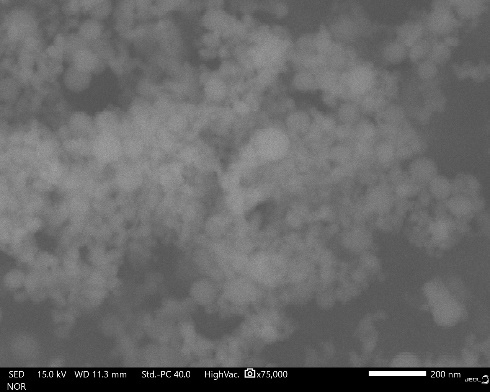

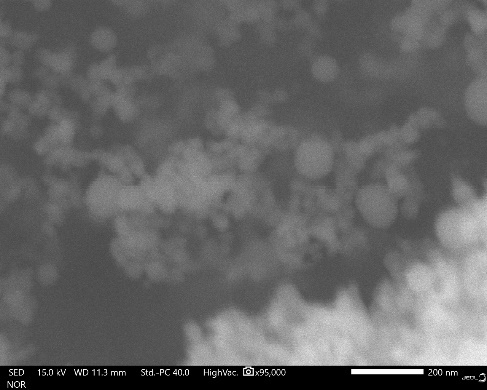


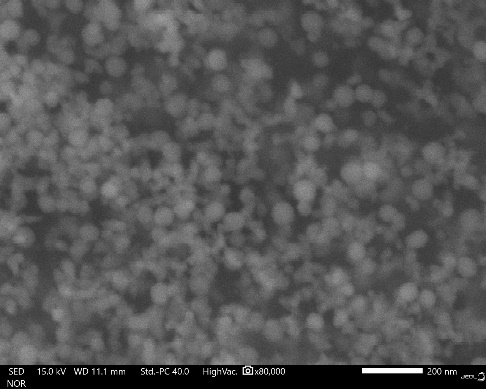

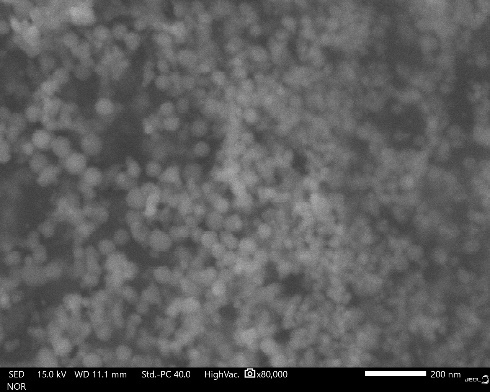


Figure S6. SEM images of PbS_x_Te_1-x_ samples synthesized with 6.4 mL N_2_H_4_ (100x) at 20°C for 60 minutes. The top images are from the top side of the composite while the bottom images are from the bottom side of the composite. The insets in some of the pictures are macroscale imaging of how the composites look.


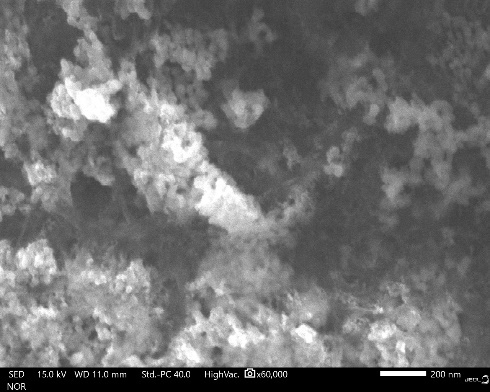

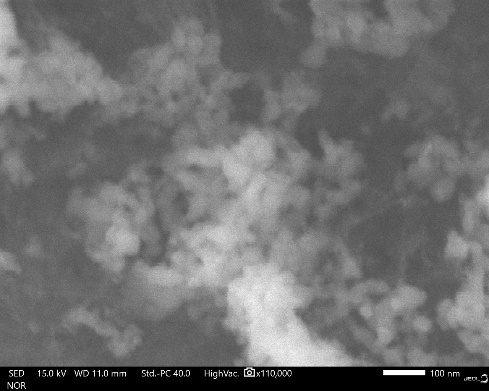

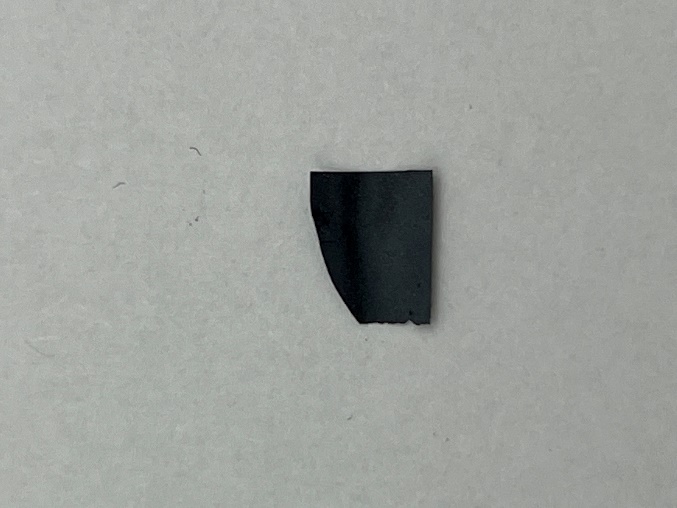

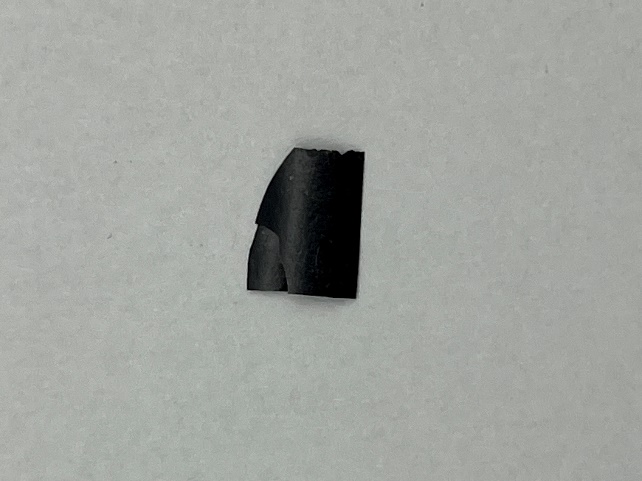


Top Bottom

Figure S7. SEM images of PbS_x_Te_1-x_ samples synthesized with 6.4 mL N_2_H_4_ (100x) at 40°C for 60 minutes. The top images are from the top side of the composite. Note that no particles are spotted via SEM from the bottom side of this composite. The bottom images are macroscale imaging of how the composites look.


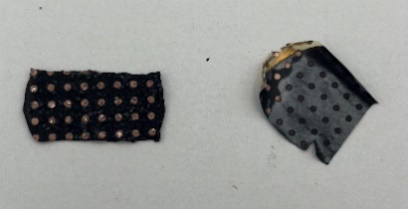

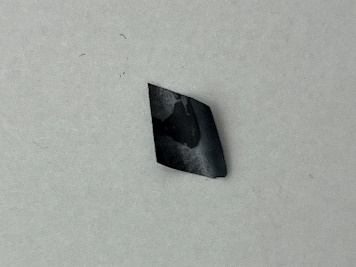

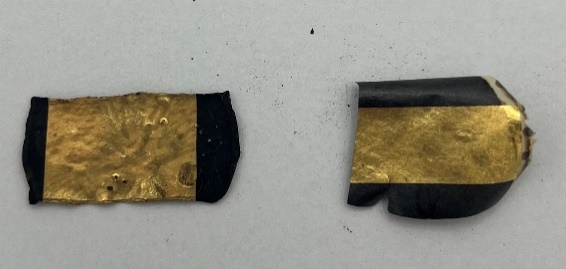

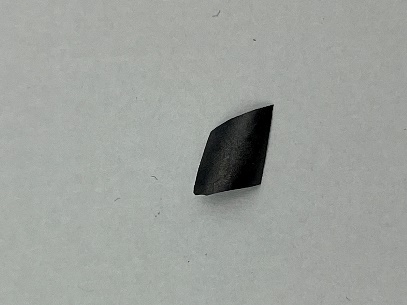

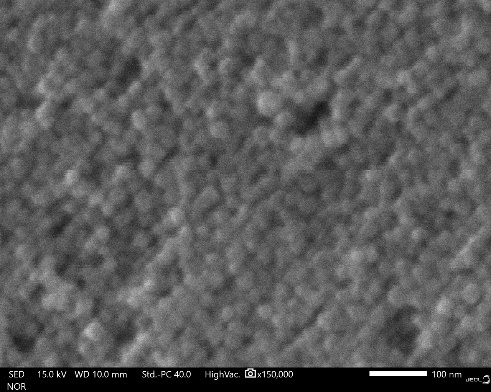

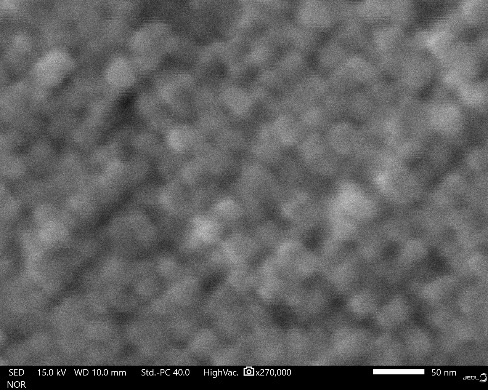

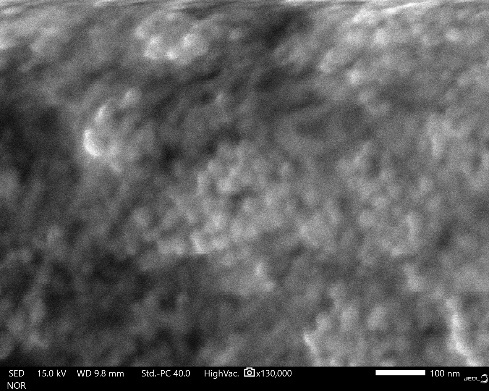

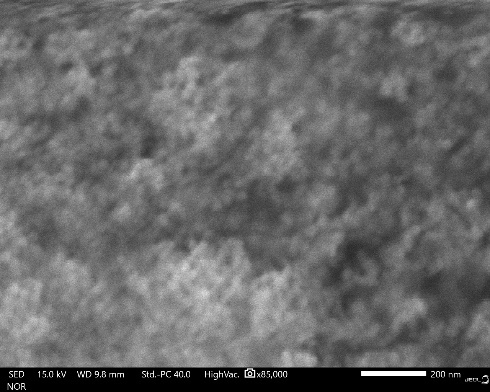


Figure S8. SEM images of PbS_x_Te_1-x_ samples synthesized with 6.4 mL N_2_H_4_ (100x) at 60°C for 60 minutes. The top images are from the top side of the composite while the bottom images are from the bottom side of the composite. On the left, the insets of the pictures are macroscale imaging of how the composites look while on the right, the insets of the pictures are those same composites with electrodes deposited on them.


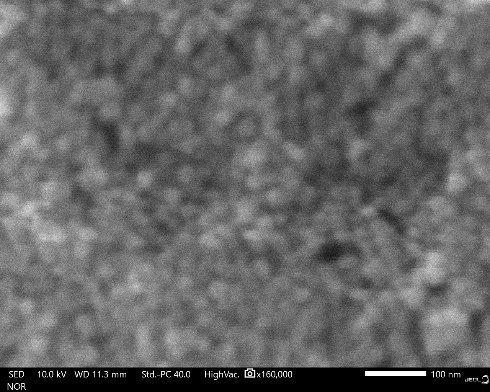

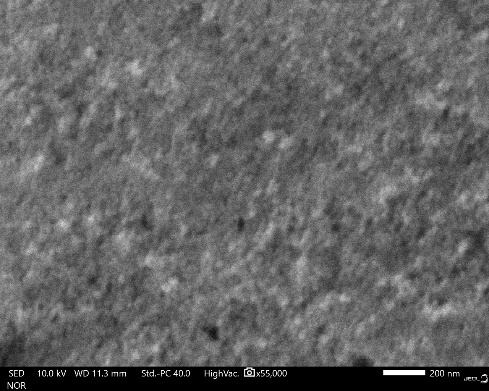


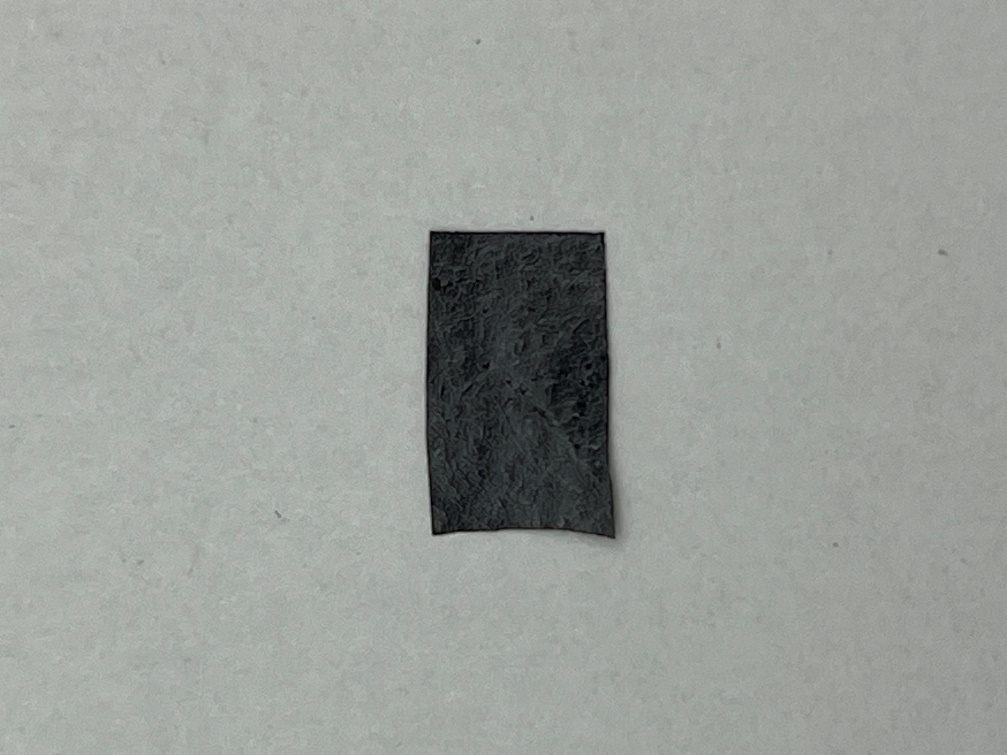

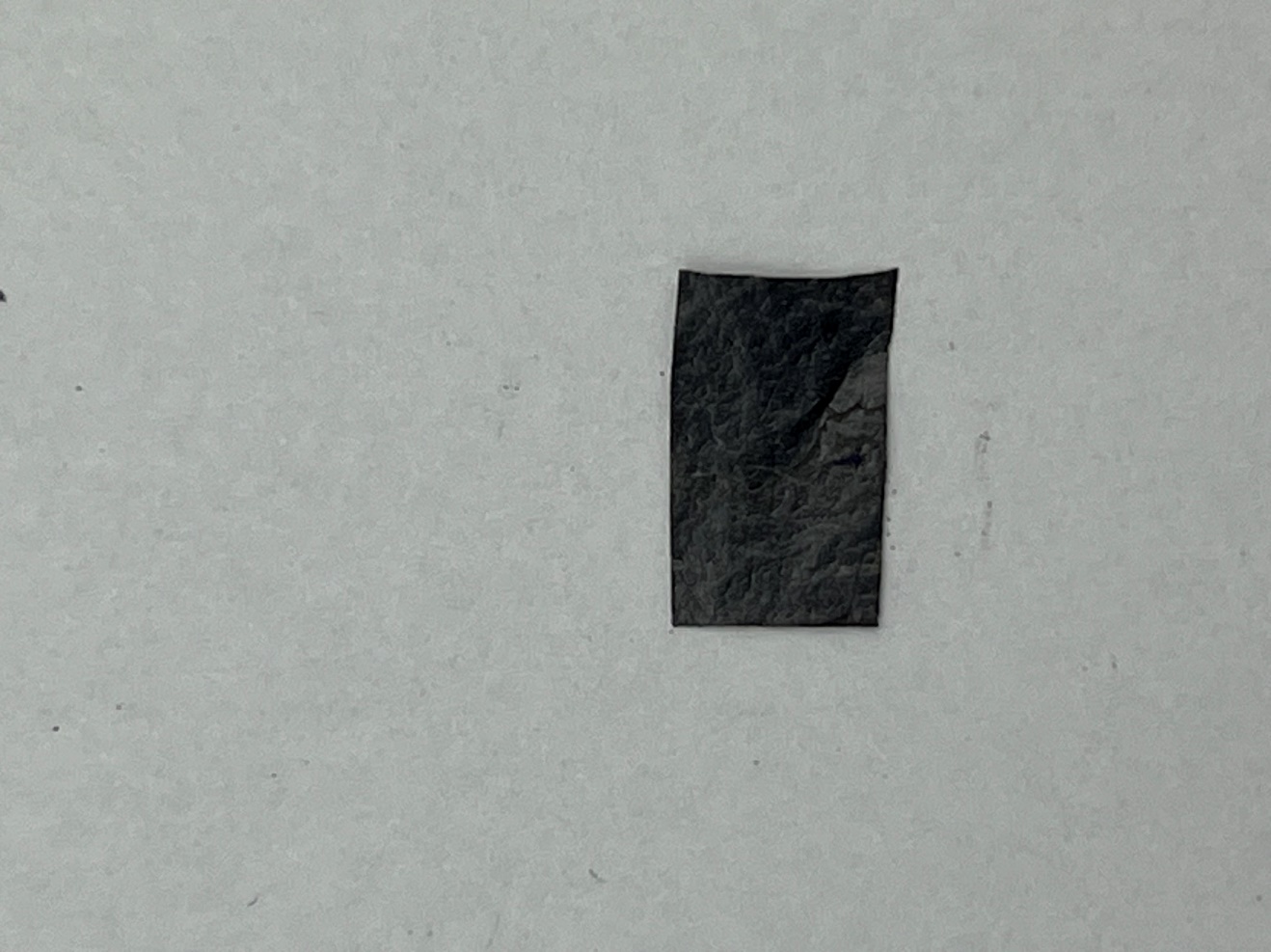


Top Bottom

Figure S9. SEM images of PbS_x_Te_1-x_ samples synthesized with 0.64 mL N_2_H_4_ (10x) at 60°C for 60 minutes. The top images are from the top side of the composite. Note that no particles are spotted via SEM from the bottom side of this composite. The bottom images are macroscale imaging of how the composites look.


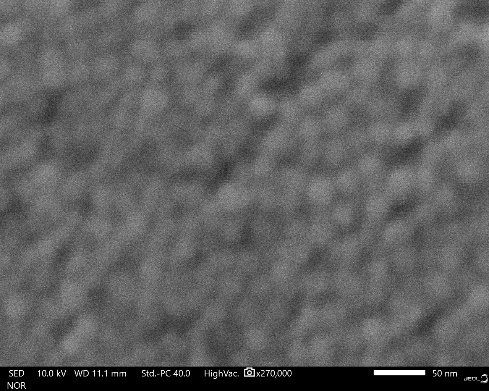

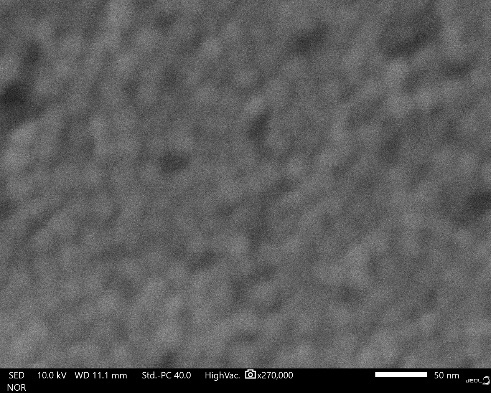


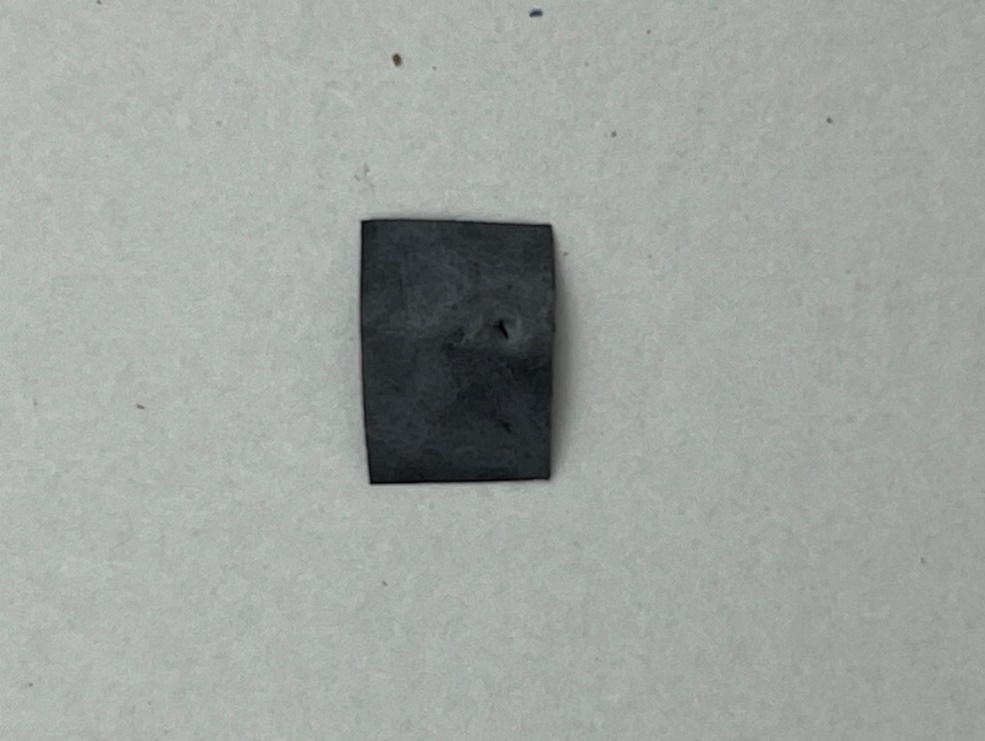

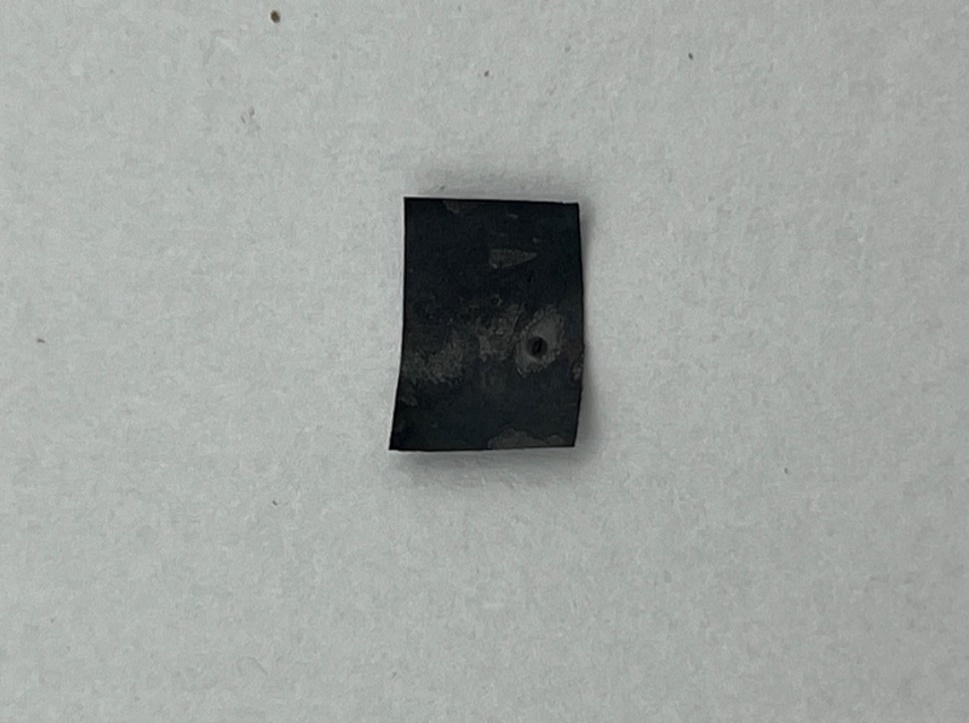


Top Bottom

Figure S10. SEM images of PbS_x_Te_1-x_ samples synthesized with 2.5 mL N_2_H_4_ (40x) at 60°C for 60 minutes. The top images are from the top side of the composite. Note that no particles are spotted via SEM from the bottom side of this composite. The bottom images are macroscale imaging of how the composites look.


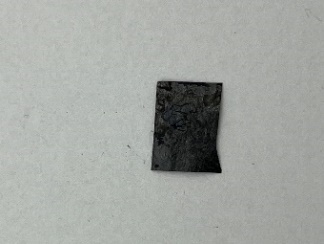

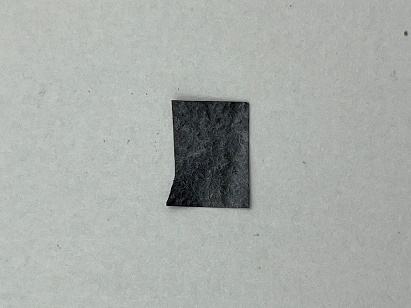

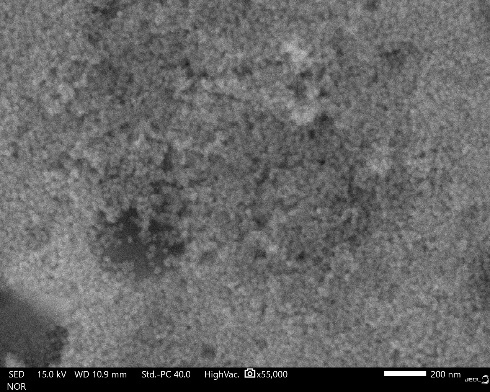

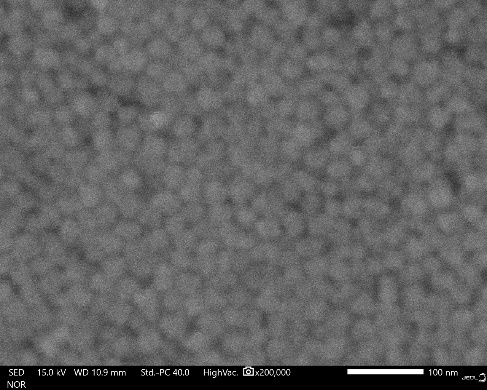

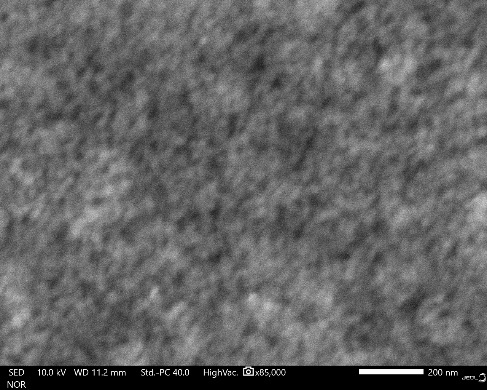

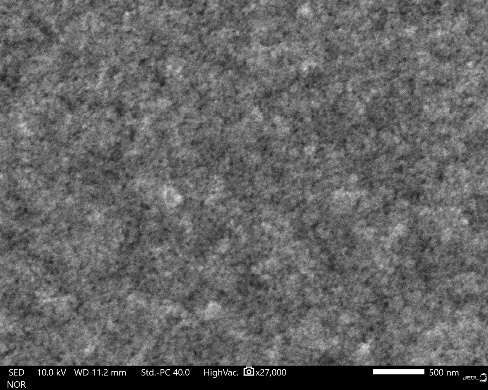


Figure S11. SEM images of PbS_x_Te_1-x_ samples synthesized with 3.5 mL N_2_H_4_ (70x) at 60°C for 60 minutes. The top images are from the top side of the composite while the bottom images are from the bottom side of the composite. The insets in some of the pictures are macroscale imaging of how the composites look.

| Element | Atom% |
| --- | --- |
| C | 47.25 |
| N | 8.70 |
| O | 9.26 |
| S | 12.06 |
| Te | 8.24 |
| Pb | 14.49 |
| Total | 100.00 |

Table S1. Elemental composition of PbS_0.8_Te_0.6_/ANF solid (density = 1.0795 g/cc) derived from SEM EDS x-ray spectra.

| Element | Line | Mass% | Atom% |
| --- | --- | --- | --- |
| C | K | 10.47 ± 0.52 | 45.03 ± 2.26 |
| O | K | 9.26 ± 0.88 | 29.91 ± 2.85 |
| S | K | 1.59 ± 0.36 | 2.56 ± 0.58 |
| Te | L | 18.55 ± 1.60 | 7.51 ± 0.65 |
| Pb | M | 60.13 ± 2.45 | 14.99 ± 0.61 |
| Total |  | 100.00 | 100.00 |

Table S2. EDS ZAF Quantification of PbS_x_Te_1-x_ samples synthesized with 6.4 mL N_2_H_4_ (100x) at 20°C for 60 minutes. Carbon can be spotted which could be from the usage of carbon paint for the imaging preparation process whereas both carbon and oxygen could be from the polyamide of the ANF, or the chain of PVP.

| Element | Line | Mass% | Atom% |
| --- | --- | --- | --- |
| C | K | 3.90 ± 0.15 | 23.90 ± 0.94 |
| O | K | 7.15 ± 0.34 | 32.92 ± 1.57 |
| S | K | 3.94 ± 0.21 | 9.05 ± 0.48 |
| Te | L | 17.50 ± 0.70 | 10.11 ± 0.40 |
| Pb | M | 67.53 ± 1.18 | 24.02 ± 0.42 |
| Total |  | 100.02 | 100.00 |

Table S3. EDS ZAF Quantification of PbS_x_Te_1-x_ samples synthesized with 6.4 mL N_2_H_4_ (100x) at 40°C for 60 minutes. Carbon can be spotted which could be from the usage of carbon paint for the imaging preparation process, the polyamide of the ANF, or the chain of PVP.

| Element | Line | Mass% | Atom% |
| --- | --- | --- | --- |
| C | K | 3.05 ± 0.12 | 25.37 ± 1.02 |
| S | K | 10.07 ± 0.23 | 31.38 ± 0.73 |
| Te | L | 4.48 ± 0.34 | 3.51 ± 0.26 |
| Pb | M | 82.40 ± 1.05 | 39.74 ± 0.51 |
| Total |  | 100.00 | 100.00 |

Table S4. EDS ZAF Quantification of PbS_x_Te_1-x_ samples synthesized with 6.4 mL N_2_H_4_ (100x) at 60°C for 60 minutes. Carbon can be spotted which could be from the usage of carbon paint for the imaging preparation process, the polyamide of the ANF, or the chain of PVP.

| Element | Line | Mass% | Atom% |
| --- | --- | --- | --- |
| C | K | 2.98 ± 0.12 | 23.01 ± 0.94 |
| O | K | 1.11 ± 0.13 | 6.44 ± 0.73 |
| S | K | 9.53 ± 0.24 | 27.51 ± 0.69 |
| Te | L | 15.86 ± 0.56 | 11.51 ± 0.41 |
| Pb | M | 70.51 ± 1.03 | 31.52 ± 0.46 |
| Total |  | 99.99 | 99.99 |

Table S5. EDS ZAF Quantification of PbS_x_Te_1-x_ samples synthesized with 6.4 mL N_2_H_4_ (100x) at 80°C for 60 minutes. Carbon can be spotted which could be from the usage of carbon paint for the imaging preparation process, the polyamide of the ANF, or the chain of PVP.


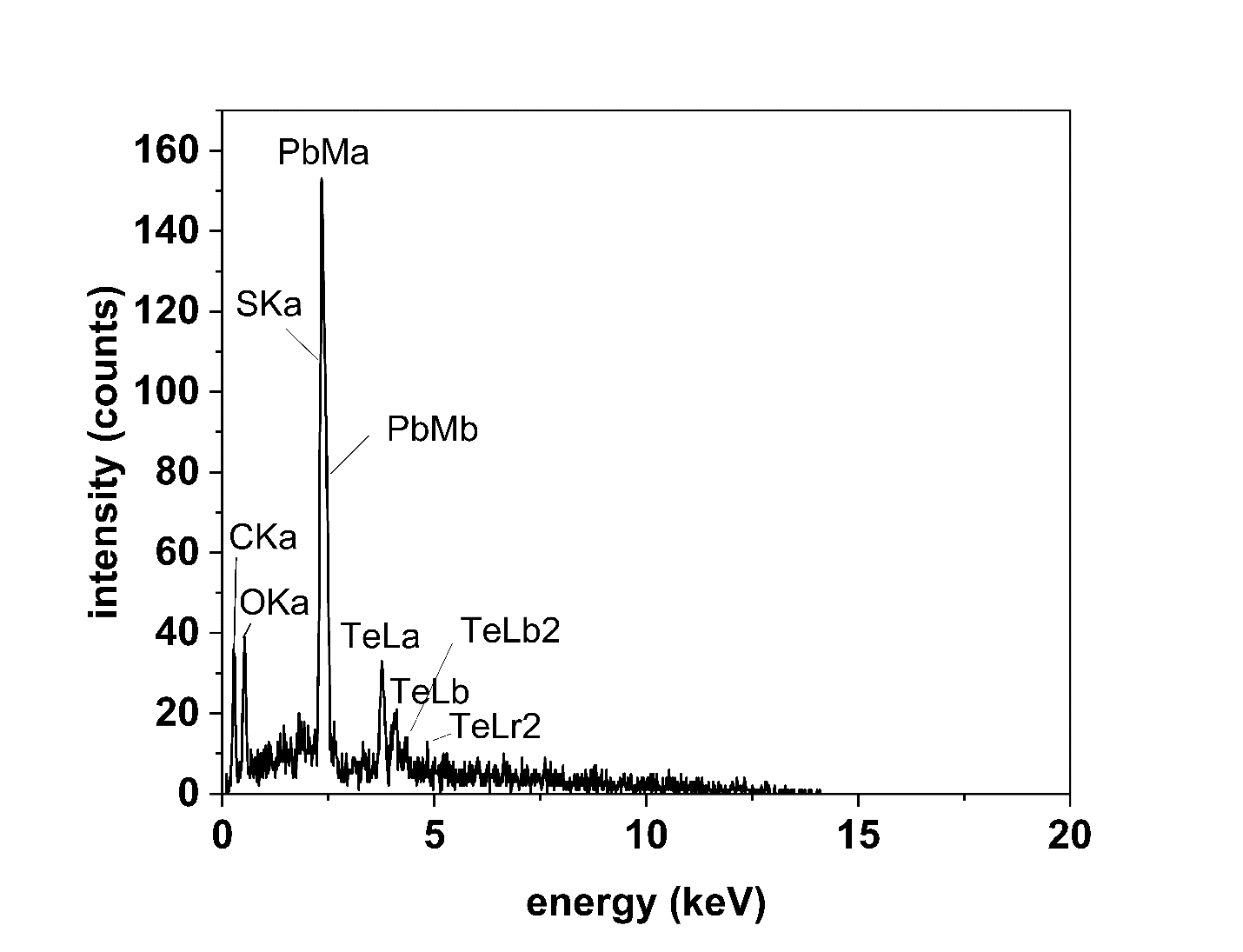


Figure S12. EDS elemental mapping of PbS_x_Te_1-x_ samples synthesized with 6.4 mL N_2_H_4_ (100x) at 20°C for 60 minutes.


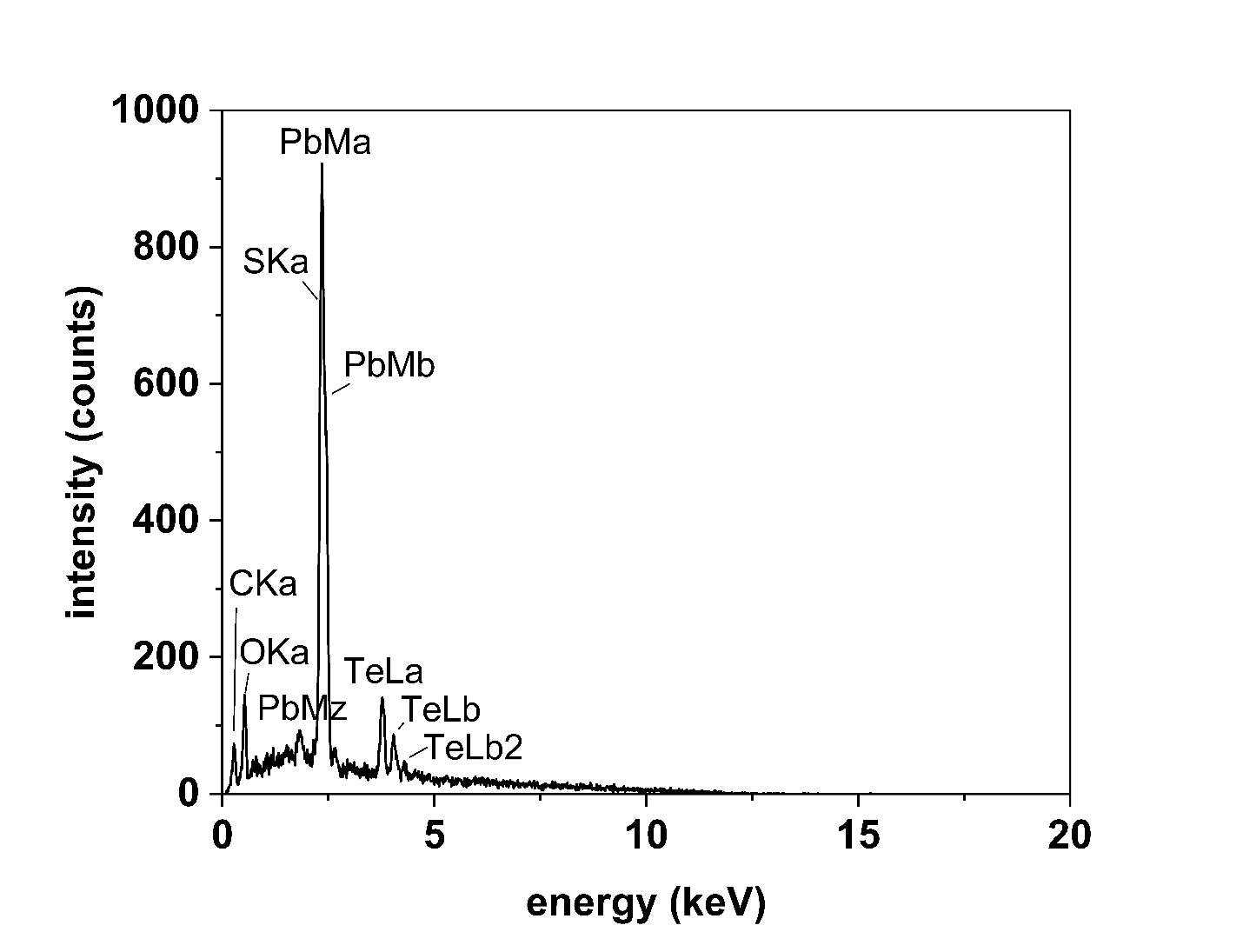


Figure S13. EDS elemental mapping of PbS_x_Te_1-x_ samples synthesized with 6.4 mL N_2_H_4_ (100x) at 40°C for 60 minutes.


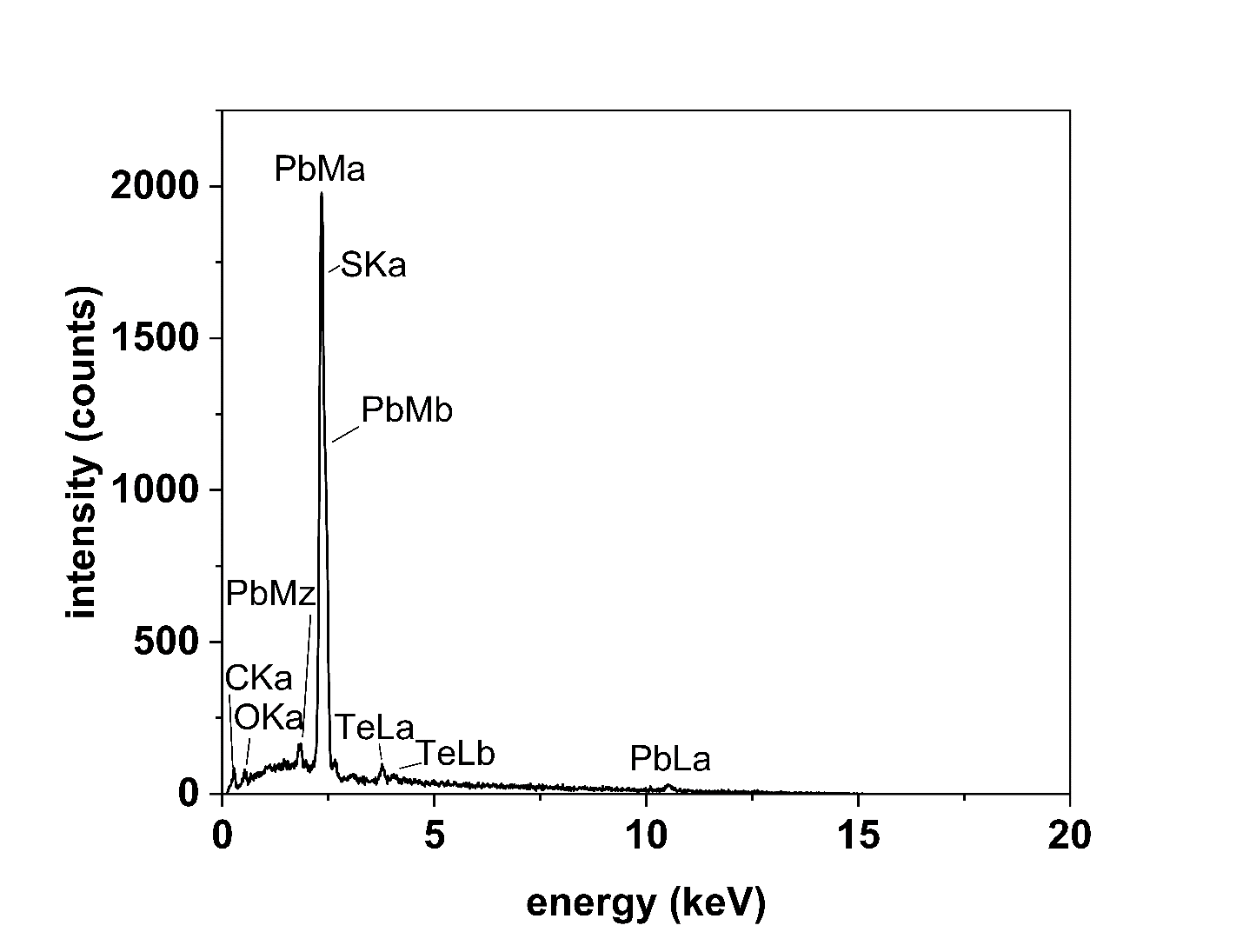


Figure S14. EDS elemental mapping of PbS_x_Te_1-x_ samples synthesized with 6.4 mL N_2_H_4_ (100x) at 60°C for 60 minutes.


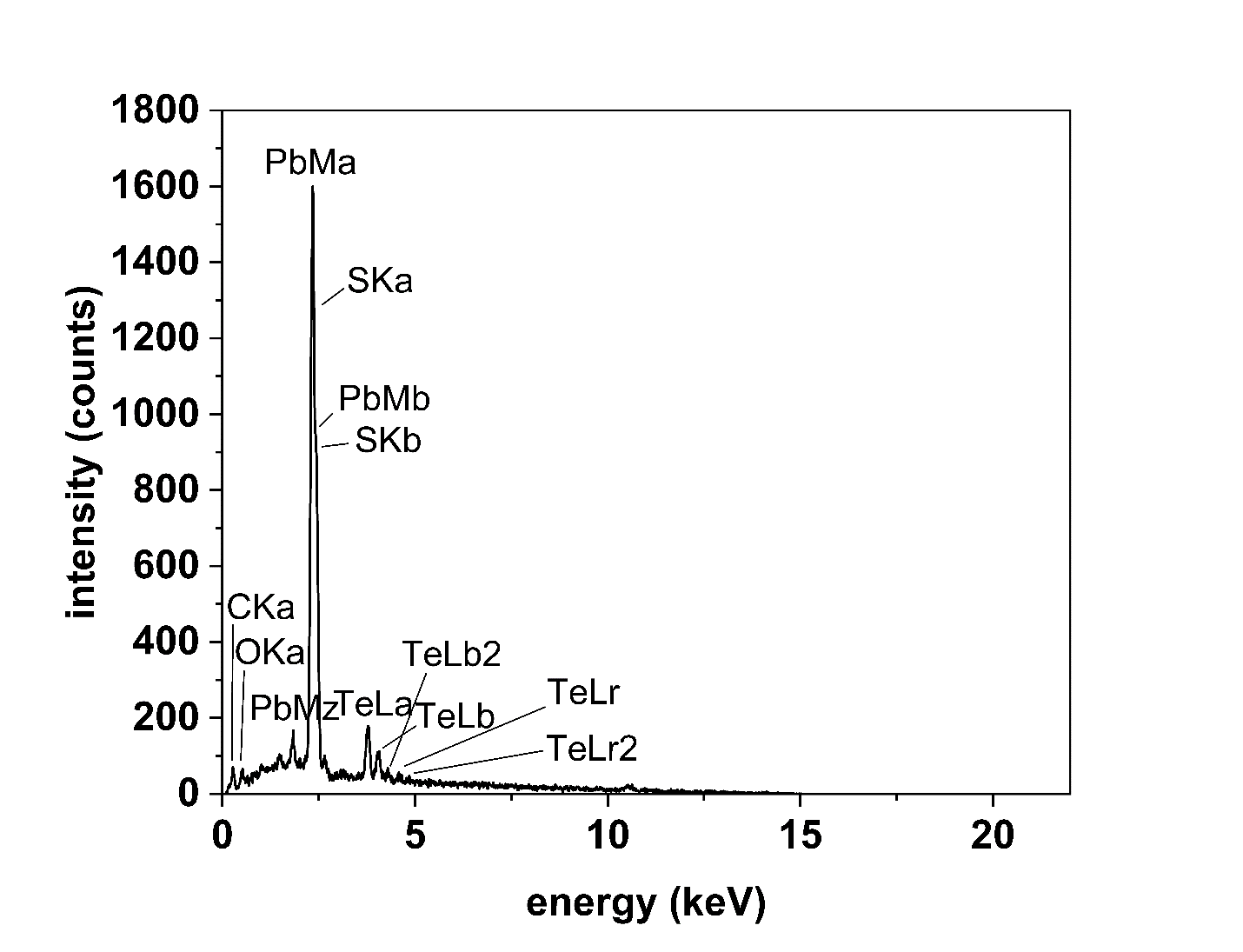


Figure S15. EDS elemental mapping of PbS_x_Te_1-x_ samples synthesized with 6.4 mL N_2_H_4_ (100x) at 80°C for 60 minutes.


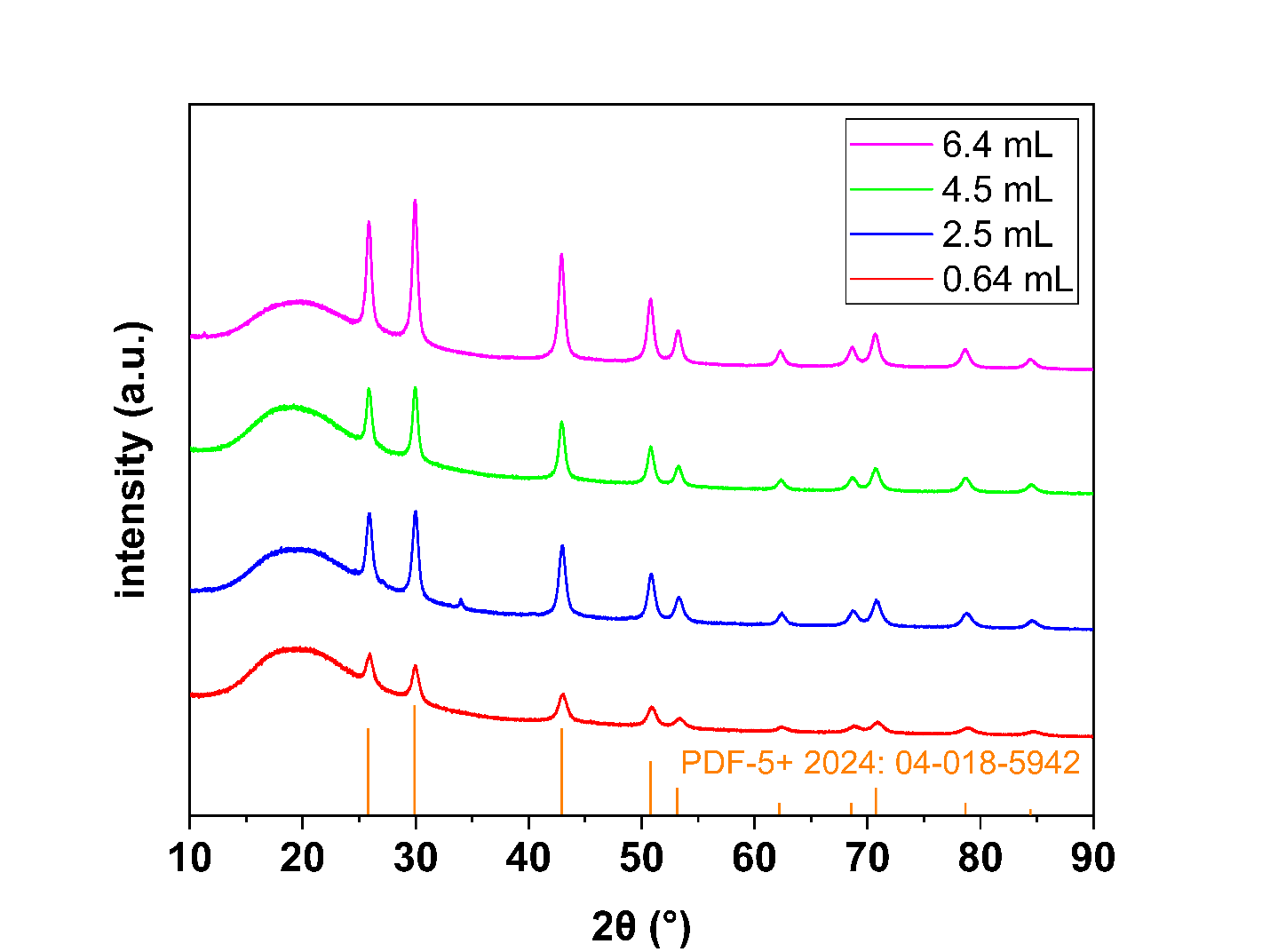


Figure S16. XRD analysis for PbS_x_Te_1-x_ synthesized at 60°C for 60 minutes at various reducing agent concentrations.


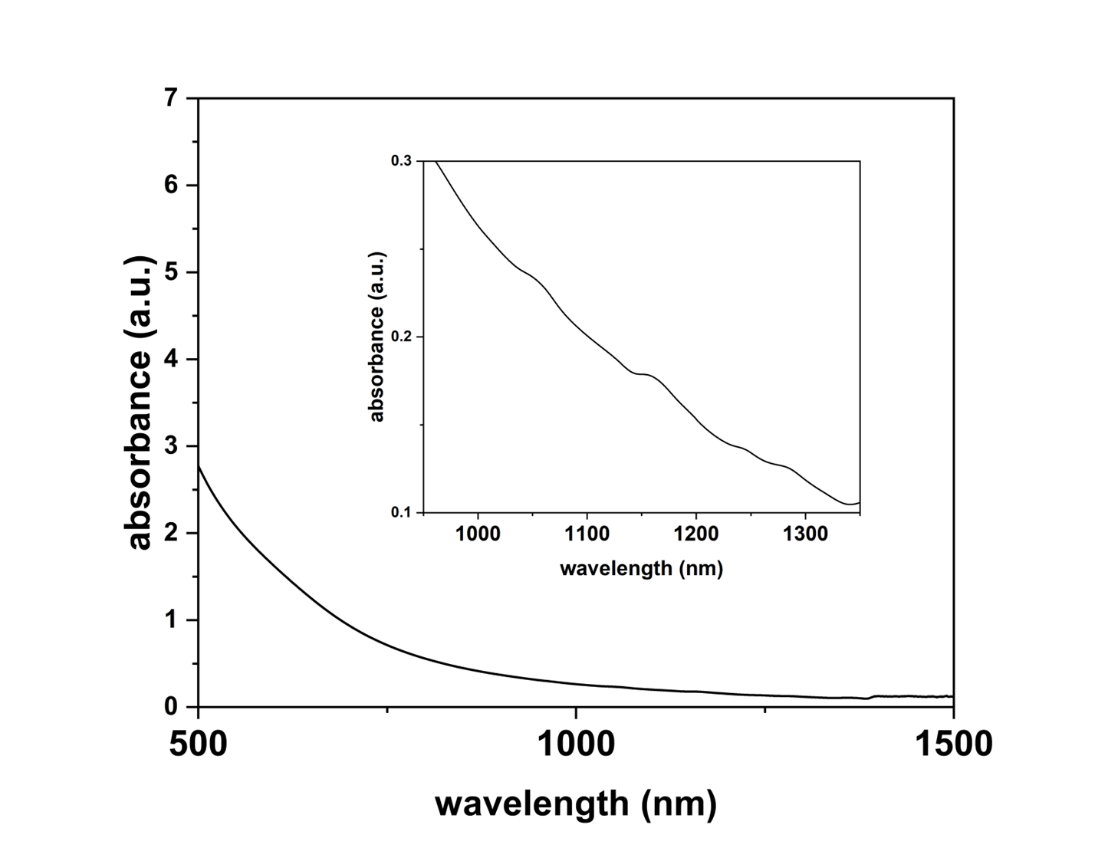


**Figure S17.** UV−vis−NIR spectrum of PbS_x_Te_y_ nanoparticles synthesized at 60°C using 6.4 mL N_2_H_4_ for 60 minutes plotted in logarithmic form for clarity.


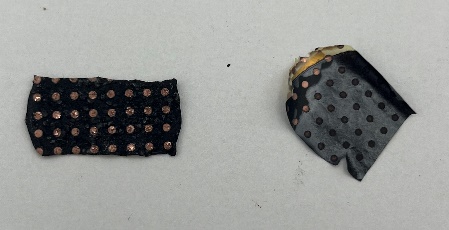

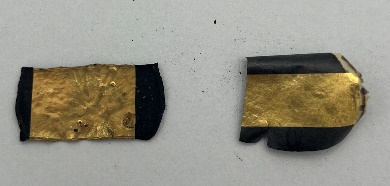

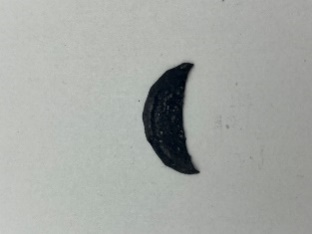

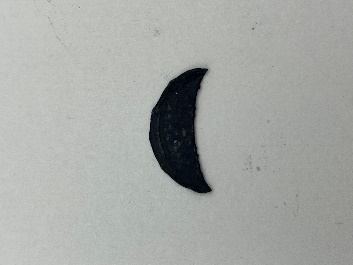

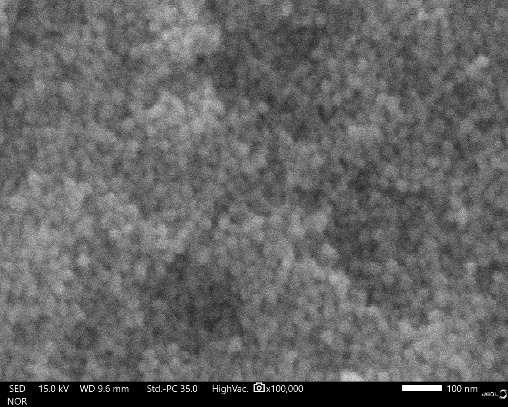

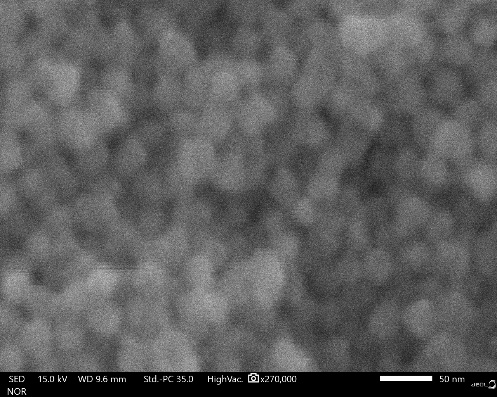


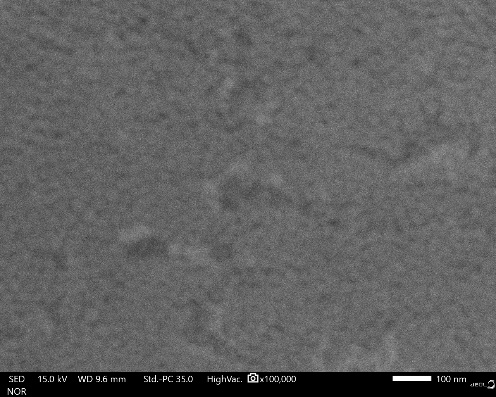

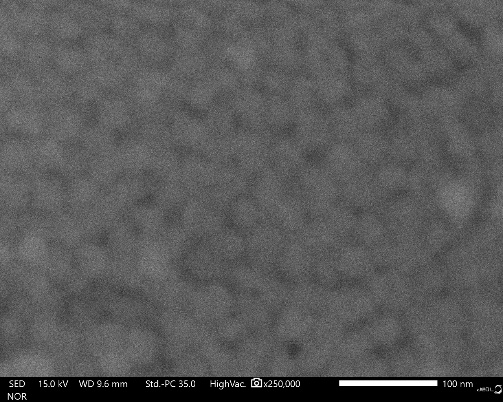


Figure S18. SEM images of PbS_0.9_Te_0.1_ samples synthesized with 6.4 mL N_2_H_4_ (100x) at 60°C for 60 minutes. The top images are from the top side of the mold ANF composite while the bottom images are from the bottom side of the composite. On the left, the insets of the pictures are macroscale imaging of how the composites look while on the right, the insets of the pictures are those same composites with electrodes deposited on them.

B

A


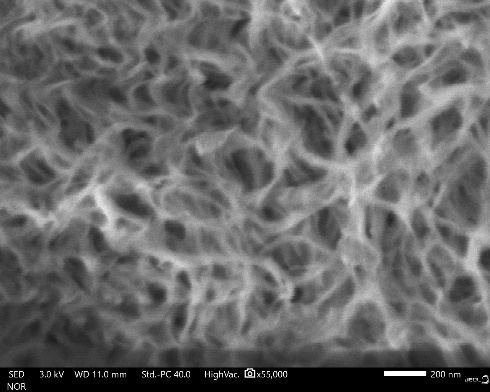

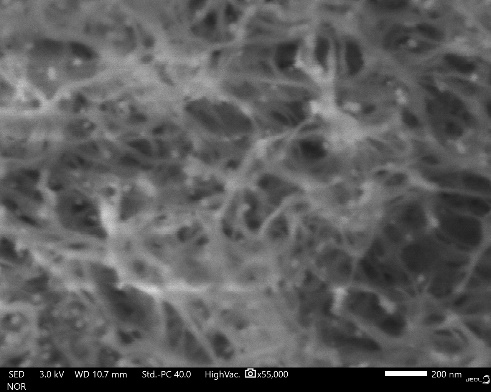


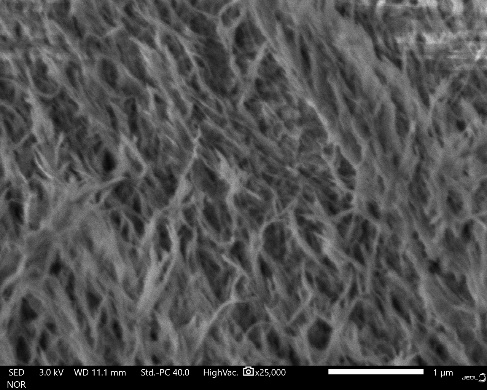

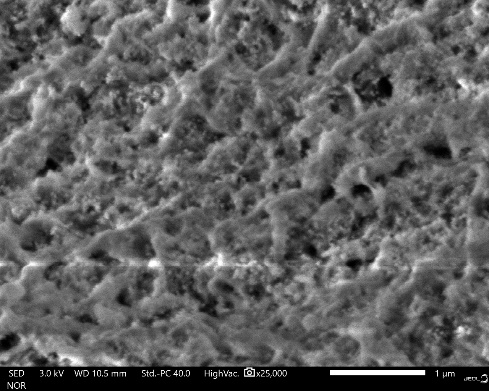


C

D

Figure S19. Cross-sectional SEM images of critical point drying (A) spin-cast thin film ANF; (B) PbS_0.9_Te_0.1_/thin film ANF composite; (C) mold ANF; and (D) PbS_0.9_Te_0.1_/thin film mold ANF composite.


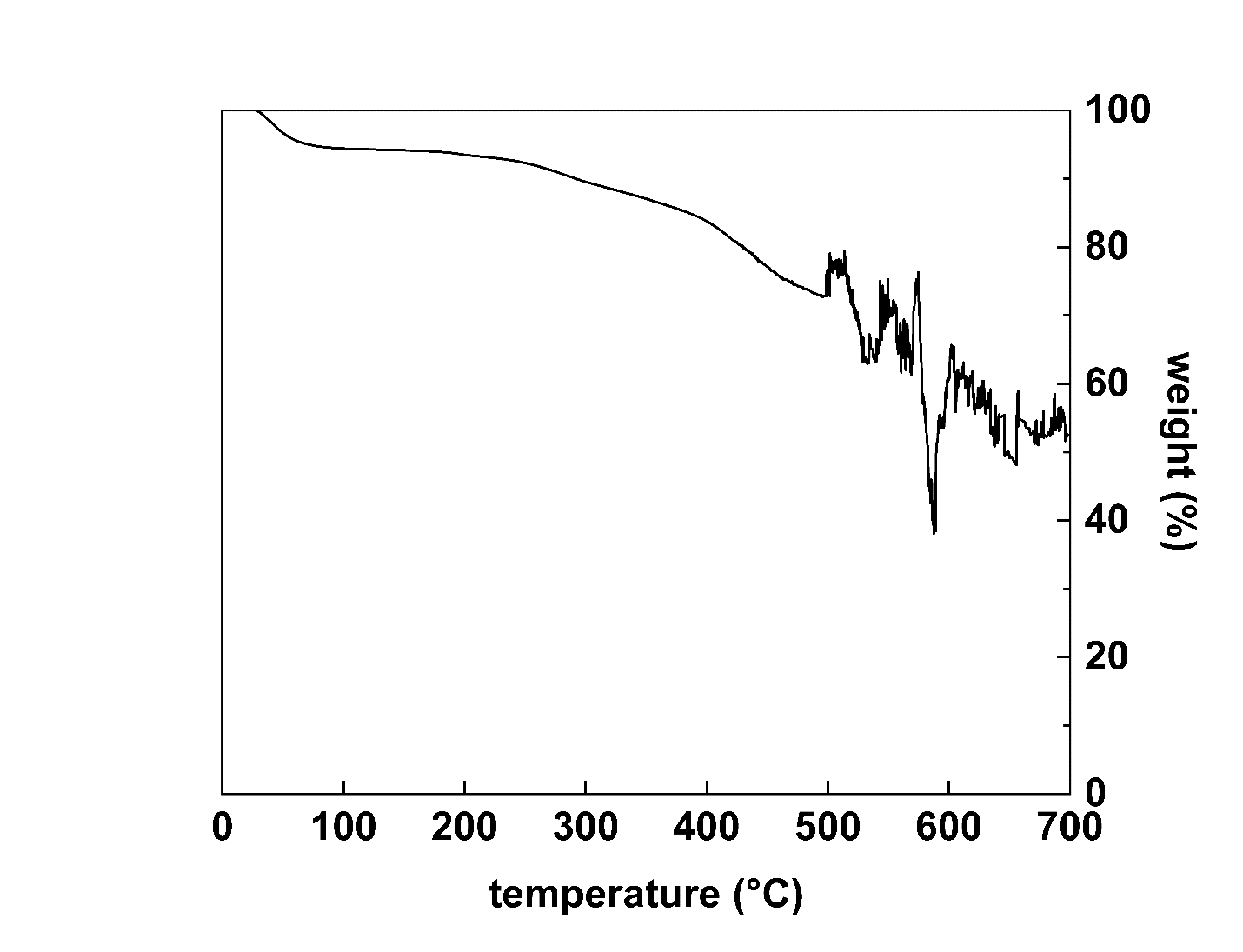


Figure S20. TGA analysis for PbS_x_Te_1-x_ samples synthesized with 6.4 mL N_2_H_4_ (100x) at 20°C for 60 minutes.


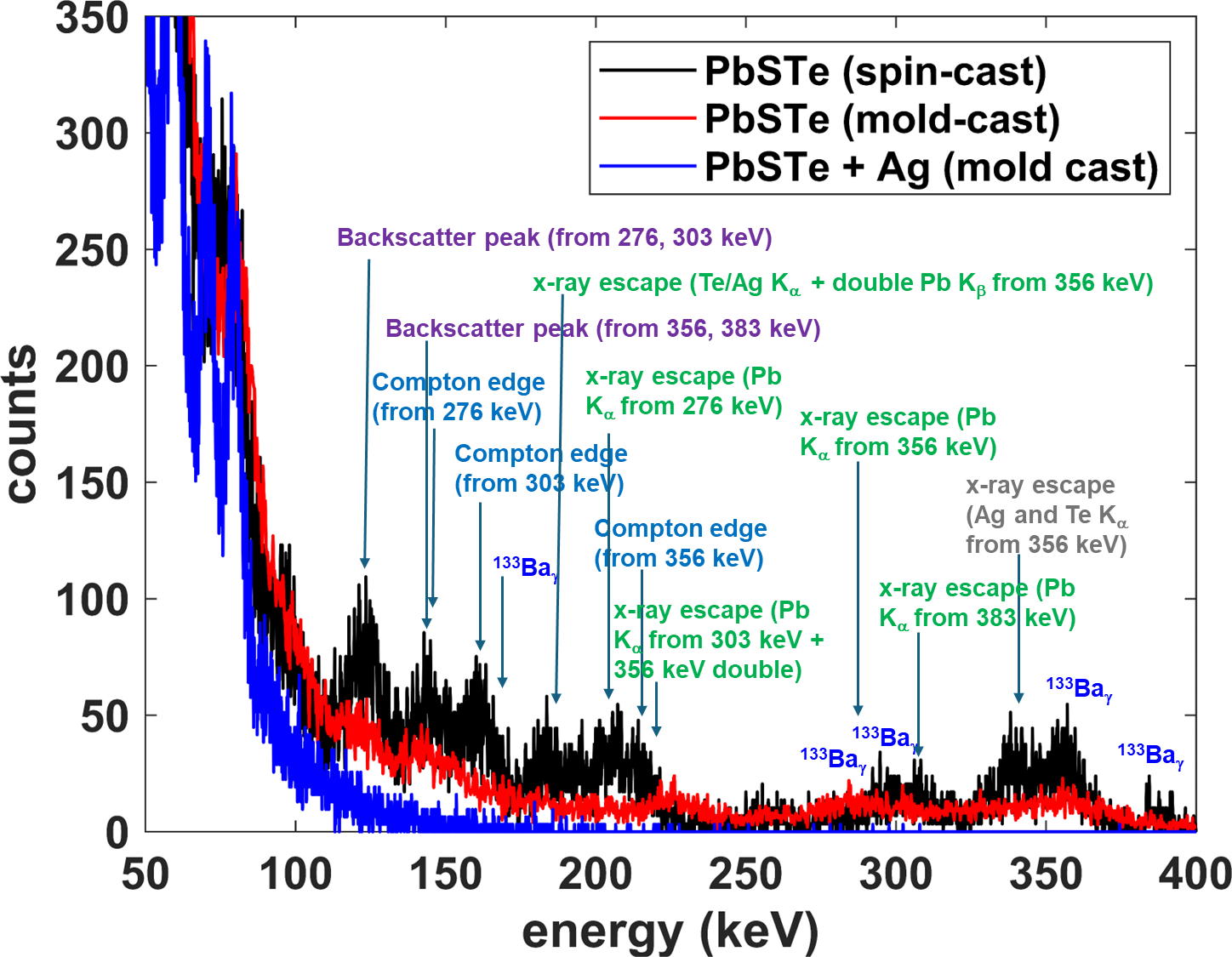


Figure S21. ^133^Ba spectra measured from: (black) PbSTe:Ag/ANF 5mm mold cast solid with 2 wt% ANF, biased to 144.8 V (leakage current 4.0 nA), (red) PbSTe /ANF 5mm mold cast 1.5 wt% ANF sample, biased to 301.0 V (leakage < 0.1 nA), (blue) Expansion of black spectra from (A). The positions of various spectral features are annotated.


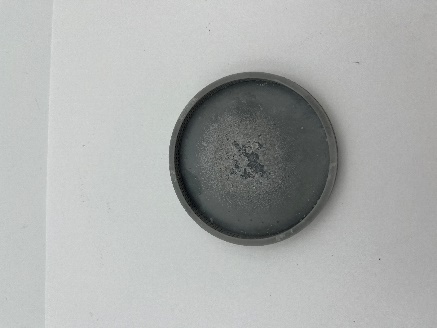

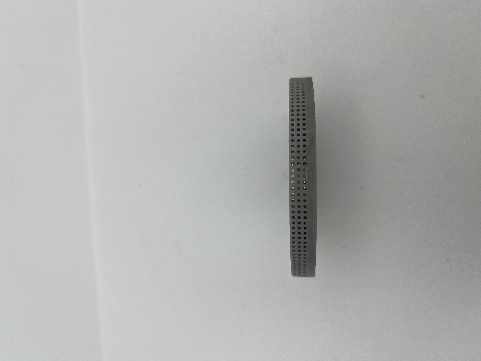


Figure S22. The 3D-printed mold for the fabrication of thick (5 mm) ANF.
